# MINDG: a drug–target interaction prediction method based on an integrated learning algorithm

**DOI:** 10.1093/bioinformatics/btae147

**Published:** 2024-03-14

**Authors:** Hailong Yang, Yue Chen, Yun Zuo, Zhaohong Deng, Xiaoyong Pan, Hong-Bin Shen, Kup-Sze Choi, Dong-Jun Yu

**Affiliations:** School of Artificial Intelligence and Computer Science, Jiangnan University, Wuxi 214122, China; School of Artificial Intelligence and Computer Science, Jiangnan University, Wuxi 214122, China; School of Artificial Intelligence and Computer Science, Jiangnan University, Wuxi 214122, China; School of Artificial Intelligence and Computer Science, Jiangnan University, Wuxi 214122, China; Department of Automation, Shanghai Jiao Tong University, Shanghai 214122, China; Department of Automation, Shanghai Jiao Tong University, Shanghai 214122, China; School of Nursing, The Hong Kong Polytechnic University, Hongkong 100872, China; School of Computer Science and Engineering, Nanjing University of Science and Technology, Nanjing 210094, China

## Abstract

**Motivation:**

Drug–target interaction (DTI) prediction refers to the prediction of whether a given drug molecule will bind to a specific target and thus exert a targeted therapeutic effect. Although intelligent computational approaches for drug target prediction have received much attention and made many advances, they are still a challenging task that requires further research. The main challenges are manifested as follows: (i) most graph neural network-based methods only consider the information of the first-order neighboring nodes (drug and target) in the graph, without learning deeper and richer structural features from the higher-order neighboring nodes. (ii) Existing methods do not consider both the sequence and structural features of drugs and targets, and each method is independent of each other, and cannot combine the advantages of sequence and structural features to improve the interactive learning effect.

**Results:**

To address the above challenges, a Multi-view Integrated learning Network that integrates Deep learning and Graph Learning (MINDG) is proposed in this study, which consists of the following parts: (i) a mixed deep network is used to extract sequence features of drugs and targets, (ii) a higher-order graph attention convolutional network is proposed to better extract and capture structural features, and (iii) a multi-view adaptive integrated decision module is used to improve and complement the initial prediction results of the above two networks to enhance the prediction performance. We evaluate MINDG on two dataset and show it improved DTI prediction performance compared to state-of-the-art baselines.

**Availability and implementation:**

https://github.com/jnuaipr/MINDG.

## 1 Introduction

Drug–target interaction refers to the binding of a drug to a specific location of target, resulting in a change in its behavior or function (Sachdev and Gupta 2019). A drug is chemical compounds which cause physiological changes in the body when consumed, injected or absorbed. A target, also known as a biological target, is a structure located in an organism that is recognized or bound by other substances such as ligands or drugs and can be acted upon by a drug or other targeted molecules (Overington *et al.* 2006). Common targets include nuclear receptors, G protein-coupled receptors, nucleic acids, enzymes, and ion channels (Landry and Gies 2008). The aim of drug–target interaction prediction is to identify novel drug compounds for biological targets and determine the therapeutic effects of drugs, which can reduce the need for complex wet experiments.

There are currently four main categories of drug–target interaction prediction methods: similarity-based methods, machine learning methods, deep learning methods, and graph learning methods.

Similarity-based methods, such as DTi2Vec (Thafar *et al.* 2021) proposed by Thafar, use Node2vec (Chen *et al.* 2020) to predict drug–target interactions. DTi2Vec maps drugs and targets to a low-dimensional vector space, preserving the similarity between nodes. These vectors can be used to predict drug–target interactions. The DTi2Vec method predicts links between drugs and proteins without mining additional internal information of drugs and proteins. Machine learning methods utilize protein structure and sequence information to predict targets. For instance, Nagamine *et al.* proposed a method that uses chemical structures, mass spectra of drugs, and amino acid sequences to represent proteins for predicting drug–target interactions (Nagamine and Sakakibara 2007). Deep learning methods combine features, models, and bioinformatics networks with other methods to achieve better prediction results. Drug–target interaction prediction involves binary classification. In contrast, drug–target binding affinity (DTA) prediction involves predicting the degree of interaction between drugs and targets as a continuous value. DTA methods provide detailed information on the interaction between drugs and targets. Öztürk *et al.* proposed DeepDTA (Öztürk *et al.* 2018), which extracts molecular features of drugs and targets separately using convolutional neural networks (Yang *et al.* 2019). Then, deep neural networks are used to predict drug–target interactions. Lee *et al.* proposed DeepConv-DTI, a deep learning method for drug–target identification (Lee *et al.* 2019). It uses deep belief networks (DBN) as a pre-processing network to pre-process drug and target features. While DeepConv-DTI is capable of obtaining local, detailed features of drugs and targets, it lacks robustness across different domains. In real-world scenarios, test and training data often come from different domains with varying distributions. To address this challenge, Abbasi *et al.* proposed the DeepCDA method (Abbasi *et al.* 2020), which is based on LSTM and CNN. Despite its good performance, DeepCDA is not effective in handling multimodal data. Dehghan *et al.* proposed TripletMultiDTI (Dehghan *et al.* 2023) to fuse multimodal knowledge for predicting interaction labels and optimizing the learning of different spatial features through the triplet loss function.

Currently, among the available methods for predicting drug–target interactions, the Graph Convolutional Network (GCN) based method shows the most promise. DTIGCCN (Shao *et al.* 2020) extracts features from the structural information of the drug and target using GCN, and then uses CNN to extract features from the sequence information of the drug and target. Wang *et al.* proposed a method for predicting drug–target interactions using the graph attention network (GAT) (Veličković *et al.* 2018, Wang *et al.* 2021) based on GCN. They conducted experiments on the Drugbank dataset (Wishart *et al.* 2018).

Intelligent computational approaches for drug–target prediction have received much attention and made significant advances (Niculescu-Mizil and Caruana, 2005)  (Brier, 1950). However, it remains a challenging task, as mentioned in the motivation. To address the challenge, this study proposes a Multi-view Integrated Learning Network (MINDG) that integrates Deep Learning and Graph Learning. The method's main principles and processes are as follows: The initial step involves processing drug–target pairs into sequence view data and structure view data. Sequence view features are then constructed using a hybrid deep network, while structure view features are constructed using a higher-order graph attention network. Finally, the multi-view features are utilized to make predictions, and the initial prediction results of each view are outputted. Finally, the initial prediction results of each view are imported into a multi-view adaptive weighted integrated decision mechanism for the final prediction. MINDG combines graph learning and deep learning to extract intrinsic structural information of drugs and proteins, as well as extrinsic relationship information between them. Therefore, our MINDG improves the performance of model prediction compared to the previous methods. However, MINDG only learns the intrinsic structural information of drugs and proteins in a sequential manner, and does not fully utilize all the intrinsic structural information available. In the future, graph learning methods may be used to learn the intrinsic structures of drugs and proteins. In addition, we have not yet conducted the wet experiment stage due to limited research. We plan to conduct further wet experiments, including drug panel test, in the future. Future research will address another issue of training drugs and targets appearing in the validation and test sets, which is a limitation of the dataset splitting.

Our contributions consist of three main aspects: (i) design an attention mechanism for the drug and protein target graph learning and propose high-order graph attention convolutional network (HOAGCN), (ii) fuse the MPNN and CNN methods to enhance the structural feature learning for drug and protein target sequences, and (iii) propose multi-view integrated learning network that integrates deep learning and graph learning (MINDG).

The rest of this study is organized as follows: Section 2 describes the specific details and principles of the proposed method in this study. Section 3 conducts an experimental study of the proposed network model, including comparisons with other methods and ablation experiments, and the experimental results are analyzed and applied. Section 4 summarizes this study and points out the shortcomings and improvement directions.

## 2 Materials and methods

**Figure 1.**
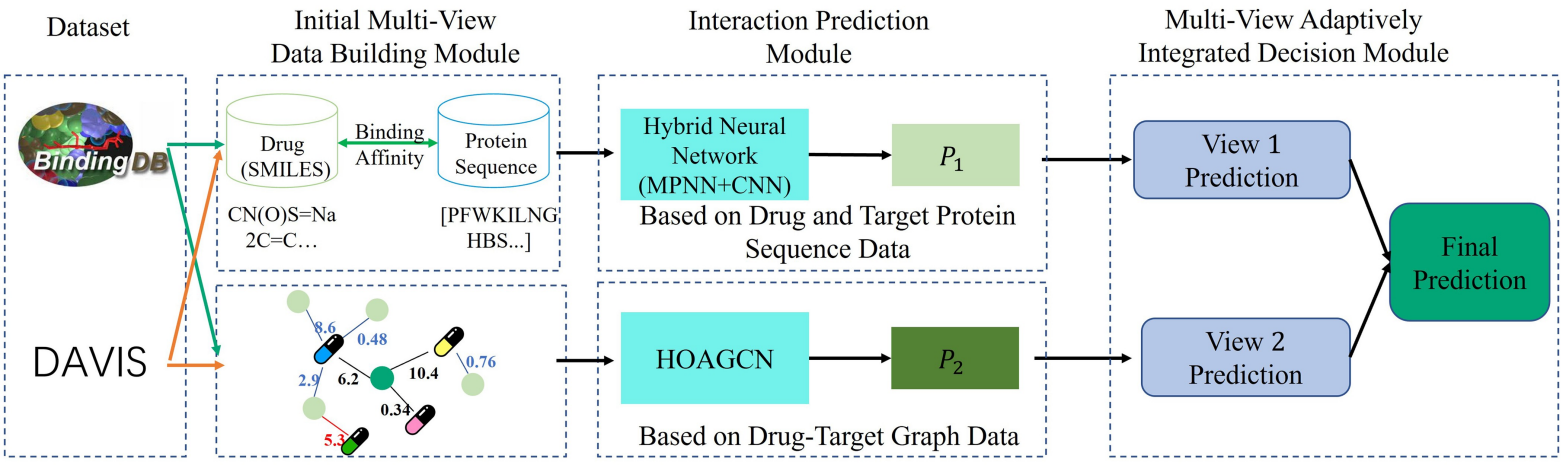
The general structure of the MINDG model proposed in this study.

The structure of the integrated learning network model proposed in this study is shown in Fig. 1. The model comprises three main modules: (i) Initial View Data Construction Module, (ii) Interaction Prediction Module, and (iii) Multi-View Adaptive Integrated Decision Module (MAIDM). These modules are briefly described in the supplementary.

### 2.1 Initial view data construction module

#### 2.1.1 DTI datasets

Our study evaluated the interaction prediction performance of MINDG using two open-source datasets: BindingDB (Liu *et al.* 2007) and DAVIS (Davis *et al.* 2011). BindingDB is a public, web-accessible database of measured binding affinities, focusing chiefly on the interactions of proteins considered to be candidate drug–targets with ligands that are small, drug-like molecules. DAVIS contains the interaction of 72 kinase inhibitors with 442 kinases, covering over 80% of the human catalytic protein kinome. Table 1 shows some information of the two datasets. We split the two datasets in a 7:1:2 ratio. The method of balancing samples is undersampling. Specific details of splitting are in Section 2.1 of the supplementary material.

**Table 1.** A brief description of the datasets used in this study.

Dataset	Nodes		DTI pairs				
	Drugs	Targets	Positive rate (%)	Training (70%)	Validation (10%)	Test (20%)	Total
Binding-DB	10 665	1413	17.01±0.01	36 599	5228	10 457	52 284
DAVIS	68	379	6.97±0.03	18 040	2577	5154	25 772

#### 2.1.2 DTI sequence view data

The binding affinity of a drug to its targets can be used to measure drug–target interactions. This affinity reflects the potency and selectivity of the drug, and is determined by the mutual attraction between the drug molecule and its target proteins. In our study, drug sequences are represented using a simplified molecular input line entry system (SMILES). Amino acid sequences are used to represent target proteins, and the labels between drugs and targets are binary values obtained by binarizing binding affinity value.

#### 2.1.3 DTI structure view data

For X drugs and Y target proteins contained in different datasets, the label between them is usually 0 or 1, or the binding affinity value. When the label is a binding affinity value, the following linkage relationship between the drug set $D=\{d_{1},d_{2},\ldots ,d_{X}\}$ and the target protein set $P=\{p_{1},p_{2},\ldots ,p_{Y}\}$ can be derived after threshold processing:

(1) Idx,py=0, I>threshlod 1, I<threshold

### 2.2 Interaction prediction module

#### 2.2.1 Hybrid deep network interaction prediction Sub-module based on sequence data

**Figure 2.**
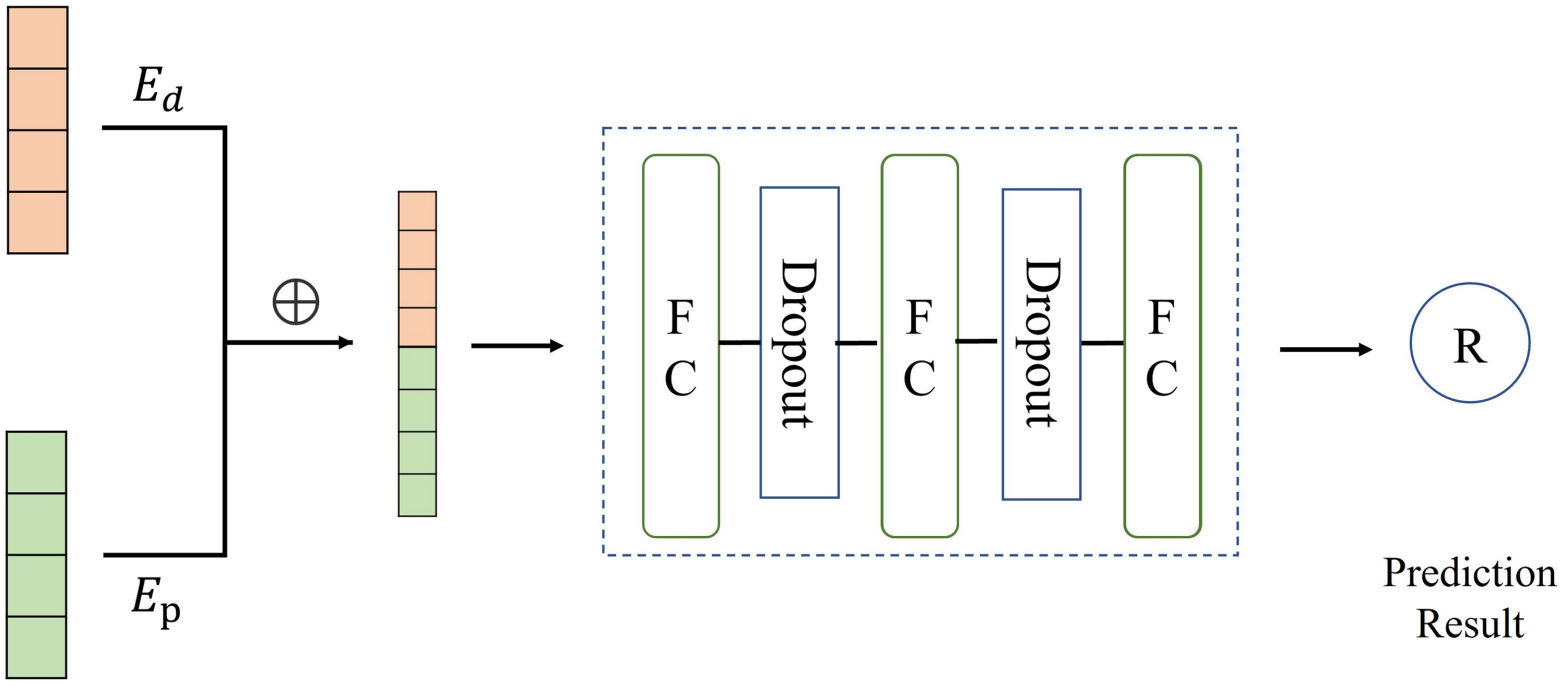
Structure diagram of the hybrid deep network (HDN).

For the sequence view, a hybrid deep network (HDN) as shown in Fig. 2 was constructed to learn the interactions between drugs and targets. The HDN is composed of two parts: the encoder and the prediction module. The encoder is made up of a Message Passing Neural Network (MPNN) (Gilmer *et al.* 2017, Shin *et al.* 2019) and a CNN (Albawi *et al.* 2017, Gu *et al.* 2018, Wu *et al.* 2019), which encode the drug sequence and the target protein sequence, respectively, to learn the interactions between drugs and targets. The drug and target's encoded features are concatenated and inputted into a prediction module that consists of fully connected layers to predict the connection probability of drug targets.

##### 2.2.1.1 Encoders for hybrid deep networks

(1) *Message passing neural network for drug sequence coding*

In this study, the MPNN was used to encode the drug, with atoms as nodes and chemical bonds as edges. The initial node features, as per Yang's method (Yang *et al.* 2019), were set to include atom type, formal charge, chirality, hybridization, aromaticity, and atomic mass. All features were encoded using One-Hot (Seger 2018), except for atomic mass which was represented by a real number. The edges' initial features include bond type, conjugation, cyclic nature, and steric effects. These features are also encoded using One-Hot. To facilitate the description of the message passing neural network, we illustrate it with an undirected graph G. Where i and j are atomic nodes in G, $x_{i}^{0}$ and $x_{j}^{0}$ are the initial features of the nodes, and $e_{ij}^{0}$ is the initial edge feature between nodes i and j.

The MPNN algorithm consists of two phases: message passing and readout. During the message passing phase, information is exchanged between atoms and node and edge features are constructed using the hidden states of nodes and edges. The readout phase utilizes these features for prediction.

The message passing phase consists of T steps, t ∈{1, …, T}. Before performing the first message delivery, Equation (2) is first used to initialize the edge hiding state $h_{ij}^{0}$ between nodes i and j:

(2) hij0=τWicatxi0,eij0

where $W_{i}\in R^{h\times h}$ is a weight matrix, $\mathrm{cat}\left(x_{i}^{0},e_{ij}^{0}\right)$ denotes the concatenation of feature $x_{i}^{0}$ and edge feature $e_{ij}^{0}$ of node i, and τ is the ReLU activation function.

On each step t, the message function $M_{t}$ and the update function $U_{t}$ are used to update the message $m_{ij}^{t}$ received by each edge and the edge hidden state $h_{ij}^{t}$.

(3) mijt+1=∑j∈NiMtxit,xjt,hijt

(4) hijt+1=Uthijt,mijt+1

In Equation (3), $m_{ij}^{t+1}$is the information received by node i at step t+1, and $N\left(i\right)$ is the set of neighboring nodes of node i. This equation indicates that the information received by the edges between nodes i and j comes from the feature $x_{i}^{t}$ of node i, the feature $x_{j}^{t}$ of neighboring nodes and the hidden state $h_{ij}^{t}$ of the edges between them. After the information is generated, it is necessary to update the hidden state of the edges. In Equation (4), $U_{t}$ is the update function, which takes the hidden state $h_{ij}^{t}$ of the edges at step t and the received message $m_{ij}^{t+1}$ as input to obtain the edge-hidden state $h_{ij}^{t+1}$ at step t+1.

In particular, the message function $M_{t}$ and the update function $U_{t}$ are defined in the form of Equations (5) and (6):

(5) Mtxit,xjt,hijt=hijt+1

(6) Uthijt,mijt+1=τhij0+Wimijt+1

After calculating the edge hidden state $h_{ij}^{t}$, it is then summed up using Equation (7) and further calculated using Equation (8) to obtain the hidden state $h_{i}$ of node i:

(7) mi=∑j∈Nihijt

(8) hi=τWicatxi,mi

In the readout phase, the hidden states of all nodes are summed to obtain the encoded features of the MPNN and are represented in Equation (9).

(9) h=∑i∈Ghi

Where in Equation (7), h is the drug encoding feature $E_{d}$ of the MPNN output.

**(2) *Convolutional neural networks for target coding***

This study employs a Convolutional Neural Network (CNN) as an encoder to encode the target protein sequences. The CNN architecture includes one or more convolutional and pooling layers. The pooling layer down-samples the output of the previous layer and generalizes the features learned by the filters. Supplementary Figure S2 in the supplementary materials illustrates the specific architecture of the CNN used in this study.

The study followed the method (Hinton *et al.* 2006) to scan 550 000 protein sequences from UniProt. Twenty classes, represented by unique letters, were extracted and each class was assigned a corresponding integer. For instance, ‘C’ was assigned 2, ‘N’ was assigned 12, ‘V’ was assigned 18, ‘S’ was assigned 16, and ‘F’ was assigned 5. The sequence ‘C N V··· S’ was encoded as [C N V ··· S] = [2 12 18 ··· 16]. The protein sequence is inputted into a 3-layer convolutional layer for convolutional operation, where the number of filters in the second layer is twice of the first layer, and the number of filters in the third layer is three times of the first layer. In this study, the number of filters is set to 32, 64, and 96, respectively. The encoded features $E_{p}$ and $E_{d}$ generated by the message passing neural network in the previous sections are concatenated and passed to the prediction unit for final prediction.

##### 2.2.1.2 Prediction unit for hybrid deep network

The prediction units of the hybrid network are three fully connected layers that receive as input the drug encoding result $E_{d}$ and the target encoding result $E_{p}$ generated by the encoder. The size of the first two FC layers is set to 1024 and each layer is followed by a Dropout of size 0.1. The dropout is a regularization technique that avoids overfitting by setting certain neurons to 0. The size of the third layer is 512, and finally the prediction results are obtained by the ReLU activation function. The prediction unit is shown in Supplementary Figure S3 in the Supplementary Materials.

#### 2.2.2 High-order graph attention convolutional network interaction prediction module based on structure data

**Figure 3.**
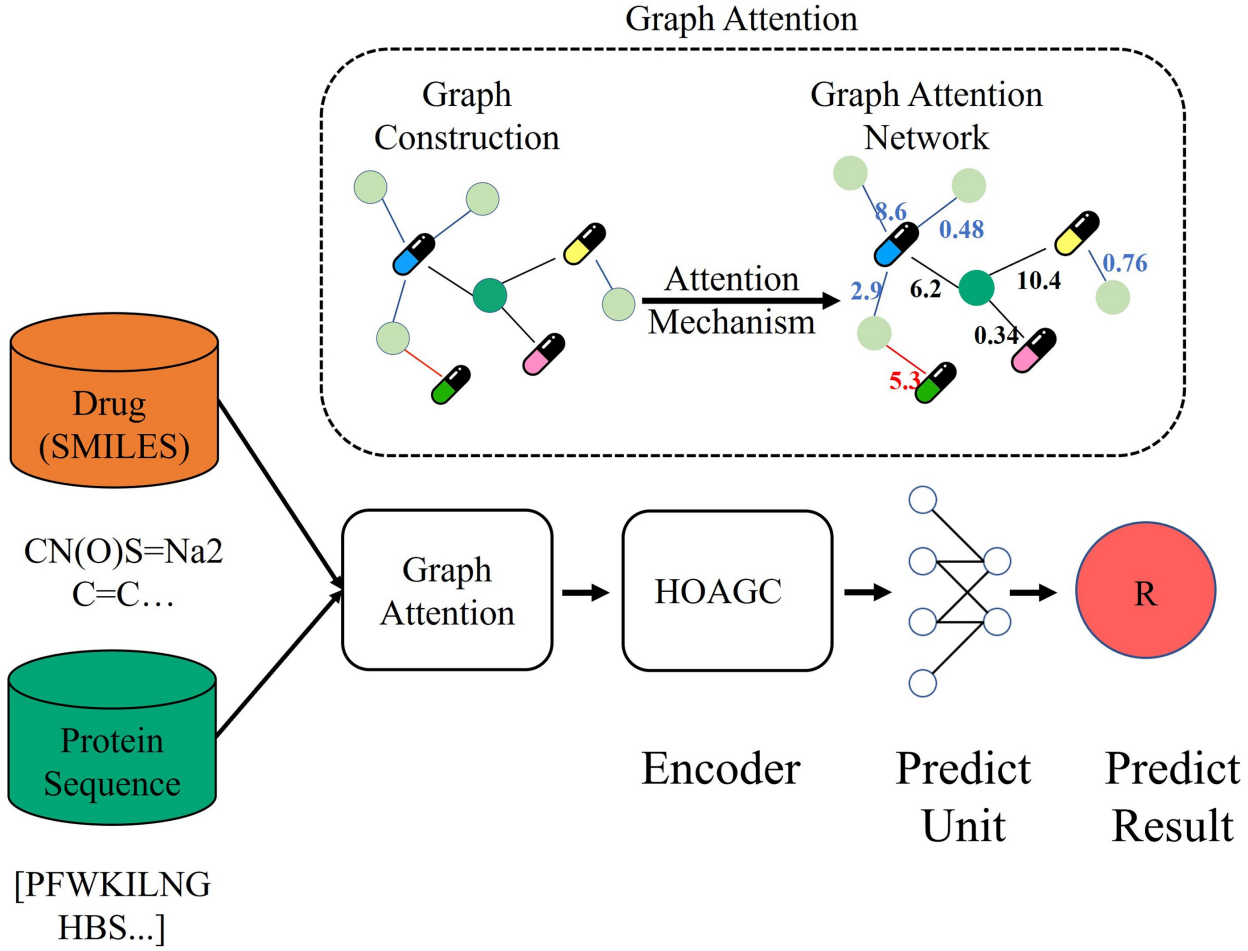
Structure of high-order graph attention convolutional network (HOAGCN).

For the drug–target relationship graph view data, a high-order graph attention convolutional network was designed to extract the structure features of drugs and targets, and the network structure is shown in Fig. 3. For the constructed drug–target structure data, the attention coefficients are assigned to the neighboring nodes by the graph attention mechanism to obtain the more important neighboring features, and then the neighboring features are aggregated by the high-order graph convolution layer to obtain the aggregated features of the nodes, and finally the drug–target interactions are predicted by the prediction unit.

The high-order graph attention network consists mainly of a graph attention network, an encoder and a prediction unit. Each module is described in the supplementary.

### 2.3 Multi-view adaptive integrated decision module (MAIDM)

The previous sections discussed the hybrid deep network prediction module and the high-order graph attention convolutional network prediction module. As different views contain varying information, their prediction results need to be fused. This study employs an adaptive weighting mechanism to fuse the losses of multiple views and construct an optimization objective function. The objective function also addresses the importance of each view. The loss function is described in Equation (10):

(10) loss=∑v=1MαvrLvZv,label

$$s.t.\;\sum_{v=1}^{M}\alpha_{v}=1,\;\alpha_{v}>0$$

where M denotes the number of views (M=2 in this study), $Z_{v}$ is the predicted drug–target interaction of the v-th view, label is the true label of the drug–target pairs, and $L_{v}$ is the cross entropy loss of the v-th view, and $\alpha_{v}$ is the fuzzy weighting coefficient of the v-th view, and r>1 is a constant that serves as the weighted fuzzy index of the v-th view. By introducing r, the weights of the views can be adaptively adjusted according to the loss of the views.

For Equation (10), when the model parameters are fixed, for the variable $\alpha_{v}$ to be optimized, the following Lagrangian function can be obtained according to the Lagrange multiplier method:

(11) Jα,λ=min∑v=1MαvrLv+ λ ∑v=1Mαv-1

where λ is the Lagrange multiplier, the derivative of J(α,λ) with respect to $\alpha_{v}$ and λ is computed, and such that it is zero, resulting in the updated weight $\alpha_{v}$ shown in Equation (13):

(12) αv=Lv11-r∑iMLi11-r

According to the above equations, the weights of each view can be adaptively adjusted during the network training process.

After obtaining the weights of each view, the weights of each view are multiplied by their respective outputs and then they are added together as the final prediction results R. This process can be represented by Equation (13):

(13) R=∑v=1Vαv⋅⁡predv

The prediction results obtained from the combined decision of the two views are expected to achieve the best prediction performance.

## 3 Results

To verify the validity of the proposed method in this study, experimental analyses were conducted in the following aspects: (i) performance comparison with current better performing drug–target interaction prediction methods; (ii) ablation experiments were conducted; and (iii) application studies of the prediction results were performed.

### 3.1 Experiment setting

#### 3.1.1 Training setting

The MINDG's learning rate is 5e−4, with batch size of 32, and training epoch of {10, 20}. All modules of MINDG are trained together, and only the trainable parameters are saved at the end of training. We provide detailed training parameters and model hyperparameters in Supplementary Tables S2 and S4 of the Supplementary Material. Experimental results are verified using the 10-fold cross-validation method (Rodriguez *et al.* 2009) with each experiment repeating 15 times to ensure that the results are statistically significant. A cryptographically secure pseudo-random number generator (CSPRNG) is used to generate 15 random seeds for each experiment.

#### 3.1.2 Metrics

To assess the performance of the proposed method, this study used seven evaluation metrics: sensitivity (Sen.), specificity (Spec.), F1-Score, Precision, Accuracy, area under the ROC curve (AUROC), and area under the PRC curve (AUPRC). All metrics have a range of [0,1], and higher values indicate better performance. The specific formula and its representation are shown in the supplementary materials.

### 3.2 Evaluation and comparison

This study compares our MINDG with three representative methods: two deep learning methods, DeepCDA (Abbasi *et al.* 2020), and TripletMultiDTI (Dehghan *et al.* 2023); and one graph neural network methods, GAT (Wang *et al.* 2021). The performance of prediction is compared using two datasets, BindingDB and DAVIS. Tables 2 and 3 present the mean and variance of 15 experiment repetitions with different random seeds. The prediction indicates the strength of the drug's binding to the protein, with 1 indicating weak binding and 0 indicating strong binding.

**Table 2.** Performance comparison of different methods on BindingDB dataset by 10-fold cross validation.

Method	AUPRC	AUROC	F1-score	Sen.	Spec.	Precision	Accuracy
DeepCDA	0.901 ± 0.012	0.894 ± 0.003	0.811 ± 0.005	0.872 ± 0.013	0.840 ± 0.004	0.760 ± 0.016	0.831 ± 0.011
TripletMulti-DTI	0.940 ± 0.003	0.931 ± 0.001	0.840 ± 0.002	0.917 ± 0.022	**0.863 ± 0.001**	0.792 ± 0.008	0.865 ± 0.005
GAT	0.923 ± 0.002	0.913 ± 0.001	0.755 ± 0.021	0.887 ± 0.001	0.851 ± 0.001	0.701 ± 0.013	0.775 ± 0.012
**MINDG**	**0.971 ± 0.008**	**0.951 ± 0.004**	**0.857 ± 0.013**	**0.923 ± 0.006**	0.842 ± 0.015	**0.800 ± 0.013**	**0.875 ± 0.007**

The bold value is the best performance of the methods in the same column.

**Table 3.** Performance comparison of different methods on DAVIS dataset by 10-fold cross validation.

Method	AUPRC	AUROC	F1-score	Sen.	Spec.	Precision	Accuracy
DeepCDA	0.909 ± 0.006	0.900 ± 0.007	0.821 ± 0.009	0.780 ± 0.008	0.913 ± 0.007	0.760 ± 0.016	0.831 ± 0.011
TripletMulti-DTI	0.964 ± 0.001	0.963 ± 0.003	0.863 ± 0.002	0.790 ± 0.001	0.945 ± 0.002	0.792 ± 0.008	0.865 ± 0.005
GAT	0.914 ± 0.001	0.904 ± 0.003	0.755 ± 0.004	0.784 ± 0.002	0.923 ± 0.002	0.701 ± 0.013	0.775 ± 0.012
**MINDG**	**0.993 ± 0.001**	**0.992 ± 0.001**	**0.896 ± 0.011**	**0.812 ± 0.005**	**0.998 ± 0.001**	**0.800 ± 0.013**	**0.875 ± 0.007**

The bold value is the best performance of the methods in the same column.

Binding a drug to a protein locally obstructs the protein's catalytic reaction with the virus. However, in reality, a drug can effectively bind to multiple proteins, resulting in a complex graph-like relationship between multiple drugs and targets. Studying the structural and relationship features of drug–protein pairs can provide expert knowledge to judge the effectiveness of unobserved pairs. The method based on both structural and relationship feature learning outperforms the method with only structural feature learning or only relational learning.

Tables 2 and 3 show the mean experimental results for all compared methods on the BindingDB and DAVIS datasets, respectively. The results are based on 10-fold cross-validation. Supplementary Tables S1 and S2 of the supplementary materials show the precise 10-fold cross-validation results of MINDG On BindingDB and DAVIS. Supplementary Tables S2 and S4 of the supplementary materials show the related model hyperparameters of MINDG on BindingDB and DAVIS.

Tables 2 and 3 show that the latest deep learning method (TripletMultiDTI) performs better than the graph learning method (GAT). Compared to GAT, TripletMultiDTI improves AUPRC and AUROC by 1.7% and 1.8% respectively on BindingDB dataset, and by 5% and 5.9% on the DAVIS dataset. Compared to the best performing models in the single method, TripletMultiDTI and GAT, MINDG improved AUPRC values by 3.1% and 4.8% respectively on BindingDB dataset, and by 2.9% and 2.9% on the DAVIS dataset. MINDG has only one metric, Spec, which is smaller than TripletMultiDTI. Despite the smaller number of samples due to undersampling in the DAVIS dataset, MINDG achieves an AUROC of 0.993 and an AUPRC of 0.992. MINDG combines the advantages of graph neural networks and deep learning methods, resulting in better performance than either method alone. MINDG utilizes graph neural networks to learn features of drug–protein relationships and deep learning methods to learn drug–protein features. The results indicate that the proposed prediction model, MINDG, performs better in predicting interactions. We used the Freidman Test method (Pereira *et al.* 2015) to test the significance of the results and the sensitivity of the binding affinity gate (He *et al.* 2017), detailed in Section 2.7 of the supplementary materials. We conducted a significance experiment of the results using the Freidman Test method (Pereira *et al.* 2015) and a sensitivity experiment of the binding affinity gates (He *et al.* 2017), detailed in Section 2.7 of the supplementary materials.

### 3.3 Ablation analysis

#### 3.3.1 The impact of multi-view learning on prediction performance

To evaluate the effectiveness of the multi-view learning mechanism, we divided the two views and MAIDM included in the method proposed in this study, and then conducted comparative experiments to determine the effectiveness of each view for multi-view learning. Specifically, let View1 denote the hybrid deep network prediction model based on the combined affinity view, View2 denote the high-order graph attention network prediction model based on the drug–target relationship graph view. -View1/2 refers to masking the effect of the result of View1/2 on the final result of the model. -MAIDM means that the Multi-View Adaptive Integrated Decision Module is not used to fuse View1 and View2, and the arithmetic mean of the view results is used as the final result output. MINDG is then compared with the three particular versions listed above. The experimental results are presented in Tables 4 and 5. As can be seen from the results, MINDG has improved metrics on all datasets compared to View1, View2, and MAIDM. The predictive ability of the corresponding model is weaker than the predictive performance based on the synergy of the two views, regardless of which individual view is used. This also indicates that multiple views have complementary roles, and through their synergy, the deep features of different views learned by the high-order graph attention network and the hybrid deep network can be more fully exploited, thus improving the performance of the overall model.

**Table 4.** Performance comparison of different MINDG views on the BindingDB dataset.

Method	AUPRC	AUROC	F1-score	Sen.	Spec.	Precision	Accuracy
**MINDG**	**0.971 ± 0.008**	**0.951 ± 0.004**	**0.857 ± 0.013**	**0.923 ± 0.006**	0.842 ± 0.015	**0.800 ± 0.013**	**0.875 ± 0.007**
−MAIDM	0.940 ± 0.007	0.932 ± 0.002	0.823 ± 0.011	0.905 ± 0.010	0.855 ± 0.001	0.786 ± 0.007	0.860 ± 0.001
−View1	0.942 ± 0.003	0.929 ± 0.002	0.844 ± 0.000	0.894 ± 0.001	0.822 ± 0.001	0.792 ± 0.013	0.869 ± 0.001
−View2	0.939 ± 0.012	0.934 ± 0.002	0.802 ± 0.021	0.913 ± 0.018	**0.889 ± 0.001**	0.781 ± 0.001	0.852 ± 0.002

The bold value is the best performance of the ablation methods in the same column.

**Table 5.** Performance comparison of different MINDG views on the DAVIS dataset.

Method	AUPRC	AUROC	F1-score	Sen.	Spec.	Precision	Accuracy
**MINDG**	**0.993 ± 0.001**	**0.992 ± 0.001**	**0.896 ± 0.011**	**0.812 ± 0.005**	**0.998 ± 0.001**	**0.998 ± 0.001**	**0.906 ± 0.011**
−MAIDM	0.968 ± 0.002	0.978 ± 0.011	0.858 ± 0.009	0.808 ± 0.003	0.965 ± 0.010	0.970 ± 0.004	0.883 ± 0.003
−View1	0.973 ± 0.003	0.986 ± 0.018	0.875 ± 0.017	0.810 ± 0.004	0.971 ± 0.019	0.982 ± 0.002	0.891 ± 0.001
−View2	0.964 ± 0.002	0.971 ± 0.001	0.842 ± 0.001	0.807 ± 0.003	0.960 ± 0.001	0.959 ± 0.005	0.875 ± 0.005

The bold value is the best performance of the ablation methods in the same column.

#### 3.3.2 The impact of multi-view adaptive integrated decision module on prediction performance

This section verifies the effectiveness of the multi-view adaptive integrated decision module used by MINDG. To evaluate its performance, we compare MINDG with the corresponding version that directly uses the simple arithmetic average of multi-view results related to Equation (14), defined as MINDG_avg. The experimental results are shown in the supplementary material (Supplementary Fig. S6).

(14) MINDG_avg=1V∑v=1VRv

### 3.4 Repurposing of antiviral drugs for COVID-19 targets

In addition, based on the target SARS-CoV-2 3CL protease of the COVID-19, we used MINDG for the exploration of antiviral drug repurposing. Using the SARS-CoV-2 3CL protease sequence resolved by Gao *et al.* (2020) input into the model, the top 10 drugs with binding affinity values were predicted as shown in Table 6. To guarantee that the top 10 drugs are not included in the training set, we utilize CD-HIT (Fu *et al.* 2012) software to quickly identify their presence. If they are detected, the drugs are excluded from the training set. Therefore, we confirm that the top 10 drugs listed in Table 6 are not part of the training set.

**Table 6.** Drug repurposing of SARS-CoV2 3CL protease.

Rank	Drug	Binding affinity
1	Foscarnet	16.365
2	Favipiravir	14.489
3	Arbidol	13.241
4	Remdesivir	13.102
5	Rimantadine	10.039
6	Rilpivirine	8.432
7	Sofosbuvir	6.891
8	Glecaprevir	6.230
9	Rimantadine	4.459
10	Amantadine	3.187

Among the predicted results in Table 5, fapiravir (Seneviratne *et al.* 2020) is currently undergoing a global multicenter clinical trial for the treatment of coronavirus. Published clinical data suggest that the drug can rapidly clear the virus and achieve relief of COVID-19 symptoms, accompanied by fewer adverse effects and higher tolerability. In February 2020, favipiravir was used in China for the experimental treatment of COVID-19 (Li and De Clercq 2020). The fourth ranked drug, remdesivir (Nhean *et al.* 2021), is a prodrug (Han and Amidon 2000, Albuquerque Silva *et al.* 2005) biotransformed into a ribonucleotide analogue inhibitor capable of inhibiting the viral RNA polymerase. Therefore, remdesivir is considered a highly promising clinical agent for the treatment of COVID-19. On 22 October 2020, the US Food and Drug Administration approved raltegravir as the first drug for the treatment of COVID-19.

The above case studies and practical applications of drug efficacy in MINDG-generated candidate drug lists help demonstrate the informative value of MINDG prediction results.

## 4 Conclusion

This study proposes an integrated learning model called MINDG, which combines a high-order graph attentional deep network and a hybrid deep network. To effectively analyze the performance of the proposed method, experiments were conducted using BindingDB and DAVIS datasets. Various comparisons were made between the proposed method and some state-of-the-art methods, and the results showed that the proposed method achieved better performance. In addition, to validate the effectiveness of this study's approach, we verified the results predicted by it using data from DrugBank. We also analyzed and applied the newly identified drug–target interactions for MINDG to explore the potential of coronavirus-targeted therapy.

## Supplementary Material

btae147_Supplementary_Data
