# Supplementary material for: MINDG: a drug–target interaction prediction method based on an integrated learning algorithm
Source: Bioinformatics. 2024 Mar 14;40(4):btae147. doi: 10.1093/bioinformatics/btae147 (PMC10997434; doi:10.1093/bioinformatics/btae147)
Supplement: btae147_Supplementary_Data [file btae147_supplementary_data.docx]

# 1. Theoretical research supplement

## 1.1 Overview

For the integrated learning network model proposed in this study, its structure is shown in Fig. S1. The model consists of three main modules and these three modules are briefly described as follows.

(1) Initial View Data Construction Module. This module constructs two view data for subsequent interaction prediction model learning: a) sequence data based on drug sequence and target protein sequence, where the label between drug targets is the binding affinity value of both, which we convert to 1 or 0 by threshold processing; b) structure data based on drug-target graph, where the label between the graph nodes (drugs and target) is 1 or 0.

(2) Interaction prediction module. It consists of the following two sub-modules: a) Hybrid deep network interaction prediction module based on sequence data. This module builds a hybrid deep network (HDN) based on message passing neural network and convolutional neural network to encode drug sequence and target protein sequence information respectively, and then concatenate the features encoded by drug and target and inputs them into the prediction unit for interaction prediction, outputs the connection probability of drug and target. b) Higher-order attention graph convolutional network interaction prediction module based on structure data. The module constructs a high-order Graph attention convolutional networks (HOGACN), which applies the attention mechanism to the high-order neighborhoods of the graph nodes to obtain the more important neighborhood features, and obtains the high-order neighborhood matrix in the graph, and then extracts the features of each node in the graph. Then feature extraction is performed for each node in the graph. In the feature extraction process, HOAGCN first calculates the features of the first-order neighbor nodes of each node in the graph, and then calculates the features of the higher-order neighbor nodes of each node in the graph through the higher-order neighborhood matrix. Finally, HOGACN classifies the connections in the graph through the full connectivity layer to predict the drug-target interactions and outputs the connection probabilities of drugs and targets.

(3) Multi-view adaptive integrated decision module. This module constructs an interaction prediction classifier with adaptive integrated decision making by weighting and aggregating the prediction results from two views through a collaborative optimization mechanism.


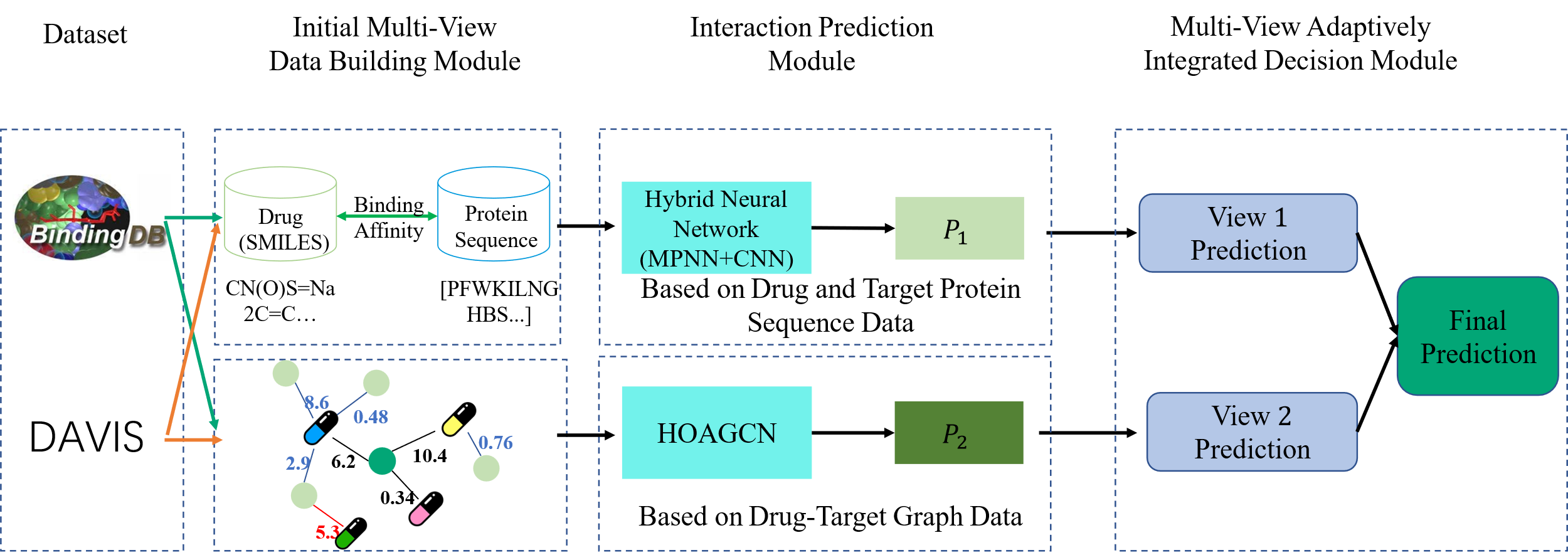


**Fig. S1.** The general structure of the MINDG model proposed in this study.

## 1.2 Related work

There are currently four main categories of drug-target interaction prediction methods: similarity-based methods, machine learning methods, deep learning methods, and graph learning methods.

Similarity-based methods assume that similar drugs or proteins have similar interaction patterns, mainly including chemical similarity, sequence similarity and network similarity. Thafar et al. (Thafar et al. 2021) proposed DTi2Vec, which uses Node2vec to predict drug-target interactions. DTi2Vec maps drugs and targets in order to preserve the similarity between nodes into a low-dimensional vector space, and these vectors can be used to predict drug-target interactions.

Machine learning methods use protein structure and sequence information to predict targets, such as Nagamine (Nagamine et al. 2007) proposed a method to predict drug-target interactions using chemical structures and mass spectra of drugs, and amino acid sequences to represent proteins. It uses SVM to classify proteins and chemicals into bound and unbound pairs, achieving accurate predictions for large amounts of data. In addition, Yu (Yu et al. 2012) uses Random Forest (RF (Biau et al. 2016)) and support vector machine for large-scale drug-target interaction prediction.

Deep learning methods combine features, models, and bioinformatics networks and other methods for drug target prediction through deep learning and achieve better prediction results. For example, DeepDTA proposed by Öztürk et al (Öztürk et al. 2018) extracts molecular features of drugs and targets separately by convolutional neural networks (Xinhua et al. 2022), and then uses deep neural networks for learning to predict drug-target interactions. DeepConv-DTI proposed by Lee et al (Lee et al. 2019) is a deep learning method for drug target identification, which uses deep belief networks (DBN ( Hinton et al. 2006)) as a pre-processing network to pre-process drug and target features. Next, the features processed by the DBN were extracted using CNN. Finally, the encoded drug and protein features are spliced and fed into a multilayer fully connected neural network for prediction. This strategy of combining multiple neural networks allows DeepConv-DTI to simultaneously consider the features of two types of sequences (drug and target protein) in different dimensions, improving the prediction accuracy and effectiveness. In addition, DeepConv-DTI further improves model prediction performance by representing drug descriptors through chemical features, combining information gain strategies to pick thresholds, and optimizing entropy power fusion. Compared with DeepDTA, DeepConv-DTI improved the AUPRC, AUROC, and F1-Score values on the DAVIS dataset by 1.2%, 0.8% and 1.6%, respectively.

Currently, among the existing drug-target interaction prediction methods, the Graph Convolutional Network (GCN (Wu et al. 2019)) based method shows better promise. Shao et al. proposed DTIGCCN ( Shao et al. 2020), which first extracts features from the structural information of the drug and the target using GCN, and then uses CNN to extract features from the sequence information of the drug and the target. Finally, these two features are combined to predict drug-target interactions. Based on GCN, Wang et al (Wang et al. 2021) proposed a method to predict drug-target interactions using graph attention network GAT (Veličković et al. 2022), and they used pseudo-position specific scoring matrix (PsePSSM) to encode target proteins and drug fingerprints to encode drugs to complete the graph construction and interaction prediction. Meanwhile, they conducted experiments on the Drugbank (Wishart et al. 2018) dataset. The experiments compared GAT with four other methods, including LRF (Shi et al. 2019), DeepConv-DTI (Lee et al. 2019), DeepDTA (Öztürk et al. 2018) and SAE+RF (Wang et al. 2018). The accuracy of DTI-GAT reached 87.67% with an AUC of 92.30%, and the prediction results were significantly higher than other existing methods, and it was relatively robust in identifying novel interactions, demonstrating the effectiveness of GAT for interaction prediction.

# 2. Experimental details supplement

## 2.1 Dataset Preprocess

The two datasets, BindingDB and DAVIS, can be found at <https://tdcommons.ai/multi_pred_tasks/dti/>. A general description of the datasets used in this study is shown in [Table 1](file:///H:\23.5.23\陈越5.23\Bioinformatics5.23\Bioinformatics-manuscript.docx#Table1). The original dataset was randomly sampled and split into training, validation, and test data subsets in a 7:1:2 ratio. A total of 52,284 samples were randomly sampled from the BindingDB dataset and split into 36,599 training samples, 5,228 validation samples, and 10,457 test samples. Likewise, A total of 25772 samples randomly sampled from DAVIS dataset were split into 18040 training samples, 2577 validation samples, and 5154 test samples. The dataset splitting method has a limitation: the drugs and targets used during training may appear in the validation and test sets. To obtain statistically significant results, we conducted each experiment 15 times using 10-fold cross-validation (Rodriguez et al. 2009). As a result, the proportion of positive and negative samples in the experiments is statistically random. Table 1 displays the related mean and variance of the positive rate.

All the samples of BindingDB and DAVIS datasets consist of five fields: Drug_ID, Drug, Target_ID, Target, and Y. Y represents the kinase dissociation constant ($K_{d}$), which reflects the interaction between drugs and protein targets. To conform with He et al. (He et al. 2017), we convert the $K_{d}$ value into a logarithmic space value, $pK_{d}$. The selected thresholds for the Davis and BindingDB datasets are 7 and 7.6, respectively. If $pK_{d}$ is greater than the threshold, the interaction of mutual attraction and binding between protein biomolecules and binding spouses is strong. Otherwise, the interaction is weak.

$$\begin{aligned} pK_{d}=-\log_{10} \left( \frac{K_{d}}{{10}^{9}} \right)\#\left（ 1 \right） \end{aligned}$$

We use the undersampling method to balance the rate of the positive and negative samples of BindingDB and DAVIS. And a large number of redundant negative samples are removed, allowing the model to focus more on learning the features of positive samples. We add the related experiments and analysis for removing redundant data in Table S5 and S6 of the supplementary material.

## 2.2 Model training and tuning

The three modules of MINDG are trained together. In the training process, MINDG model dynamically fuses the results of the two views through the fusion module, and calculates the loss between the predicted results and the ground true.

For extreme fine-tuning, the initial value of the learning rate is 5e-4, and the scheduling algorithm uses StepLR. Every 10 epoches, the learning rate is adjusted. The weights about the fusion of the two views are trainable, with initial values of {0.9,0.1}, others can also successfully converge, but more rounds are needed. The training round determination, is determined according to the samples contained in the training dataset, the more knowledge contained, the more training rounds are needed, and we choose {5,10,20} in the experiment. As for the selection of training batch size, it is easier to extract common features between samples when the batch size becomes larger. In the experiment, we used {32,64,128} and achieved good performance, and in the final formal experiment, {32} was used.

Table S1. 10-fold cross-validation results for MINDG on BindingDB

| Fold | AUPRC | AUROC | F1-Score | Sen. | Spec. | Precision | Accuracy |
| --- | --- | --- | --- | --- | --- | --- | --- |
| 1 | 0.957 | 0.954 | 0.837 | 0.931 | 0.828 | 0.800 | 0.874 |
| 2 | 0.965 | 0.952 | 0.854 | 0.920 | 0.859 | 0.796 | 0.871 |
| 3 | 0.969 | 0.951 | 0.877 | 0.926 | 0.823 | 0.811 | 0.865 |
| 4 | 0.962 | 0.953 | 0.846 | 0.909 | 0.852 | 0.779 | 0.876 |
| 5 | 0.977 | 0.955 | 0.849 | 0.933 | 0.829 | 0.812 | 0.881 |
| 6 | 0.973 | 0.948 | 0.859 | 0.923 | 0.831 | 0.813 | 0.877 |
| 7 | 0.971 | 0.953 | 0.873 | 0.931 | 0.844 | 0.803 | 0.87 |
| 8 | 0.982 | 0.953 | 0.866 | 0.929 | 0.835 | 0.793 | 0.869 |
| 9 | 0.970 | 0.955 | 0.836 | 0.917 | 0.849 | 0.823 | 0.877 |
| 10 | 0.964 | 0.946 | 0.860 | 0.924 | 0.843 | 0.798 | 0.872 |
| Final | 0.971±0.008 | 0.951±0.004 | 0.857±0.013 | 0.923±0.006 | 0.842±0.015 | 0.800±0.013 | 0.875±0.007 |

Table S2. Hyperparameters of MINDG on BindingDB

|  | parameter name | value |
| --- | --- | --- |
| Training | train epoch | 20 |
|  | batch size | 32 |
|  | learning rate | 5.00E-04 |
| View1（HDN） | fuzzy weighting coefficient | 0.9 |
|  | drug_encoding | "MPNN" |
|  | target_encoding | "CNN" |
|  | cls_hidden_dims | [1024,1024,512] |
|  | hidden_dim_drug | 128 |
|  | mpnn_hidden_size | 128 |
|  | mpnn_depth | 3 |
|  | cnn_target_filters | [32,64,96] |
|  | cnn_target_kernels | [4,8,12] |
| View2（HOAGCN） | fuzzy weighting coefficient | 0.1 |
|  | class_num | 1 |
|  | layers_1_dims | [32, 32, 32, 32] |
|  | layers_2_dims | [32, 32, 32, 32] |
|  | hidden1_size | 64 |
|  | hidden2_size | 32 |
|  | dropout | 0.1 |

Table S3. 10-fold cross-validation results for MINDG on DAVIS

| Fold | AUPRC | AUROC | F1-Score | Sen. | Spec. | Precision | Accuracy |
| --- | --- | --- | --- | --- | --- | --- | --- |
| 1 | 0.995 | 0.993 | 0.923 | 0.800 | 0.988 | 0.779 | 0.882 |
| 2 | 0.994 | 0.994 | 0.908 | 0.811 | 0.989 | 0.803 | 0.867 |
| 3 | 0.992 | 0.991 | 0.884 | 0.818 | 0.988 | 0.790 | 0.883 |
| 4 | 0.995 | 0.991 | 0.875 | 0.810 | 0.987 | 0.787 | 0.878 |
| 5 | 0.994 | 0.991 | 0.907 | 0.818 | 0.988 | 0.778 | 0.874 |
| 6 | 0.992 | 0.993 | 0.882 | 0.820 | 0.986 | 0.792 | 0.874 |
| 7 | 0.993 | 0.992 | 0.922 | 0.812 | 0.988 | 0.795 | 0.873 |
| 8 | 0.992 | 0.991 | 0.897 | 0.819 | 0.990 | 0.801 | 0.876 |
| 9 | 0.992 | 0.992 | 0.891 | 0.810 | 0.989 | 0.795 | 0.863 |
| 10 | 0.993 | 0.990 | 0.880 | 0.814 | 0.988 | 0.795 | 0.879 |
| Final | 0.993±0.001 | 0.992±0.001 | 0.896±0.011 | 0.812±0.005 | 0.998±0.001 | 0.800±0.013 | 0.875±0.007 |

Table S4. Hyperparameters of MINDG on DAVIS

|  | parameter name | value |
| --- | --- | --- |
| Training | train epoch | 10 |
|  | batch size | 32 |
|  | learning rate | 5.00E-04 |
| View1（HDN） | fuzzy weighting coefficient | 0.8 |
|  | drug_encoding | "MPNN" |
|  | target_encoding | "CNN" |
|  | cls_hidden_dims | [1024,1024,512] |
|  | hidden_dim_drug | 128 |
|  | mpnn_hidden_size | 128 |
|  | mpnn_depth | 3 |
|  | cnn_target_filters | [32,64,96] |
|  | cnn_target_kernels | [4,8,12] |
| View2（HOAGCN） | fuzzy weighting coefficient | 0.2 |
|  | class_num | 1 |
|  | layers_1_dims | [32, 32, 32, 32] |
|  | layers_2_dims | [32, 32, 32, 32] |
|  | hidden1_size | 64 |
|  | hidden2_size | 32 |
|  | dropout | 0.1 |

## 2.3 Influence on the model’s performance

In order to illustrate the impact of redundant data on the performance of the model, we gradually delete redundant samples on the experimental datasets to test the performance of MINDG. In Tables S1 and S2, p represents the number of positive samples and n represents the number of negative samples (redundant data). From these two tables, we can see that as the number of redundant data gradually decreases, the model's index increases, which means that the performance of the model becomes better.

Table S5. The performance of different data preprocessing method on BindingDB dataset by undersampling

| Samples | AUPRC | AUROC | F1-Score | Sen. | Spec. | Precision | Accuracy |
| --- | --- | --- | --- | --- | --- | --- | --- |
| p=7,843  n=44,441  (original) | 0.917±0.013 | 0.910±0.004 | 0.596±0.005 | 0.901±0.012 | 0.321±0.015 | 0.360±0.031 | 0.591±0.026 |
| p'=p, n'=0.88n | 0.907±0.007 | 0.901±0.002 | 0.400±0.005 | 0.910±0.004 | 0.250±0.012 | 0.312±0.023 | 0.469±0.025 |
| p'=p, n'=0.71n | 0.943±0.009 | 0.817±0.007 | 0.440±0.007 | 0.901±0.008 | 0.490±0.008 | 0.513±0.031 | 0.496±0.025 |
| p'=p n'=0.53n | 0.948±0.004 | 0.869±0.007 | 0.592±0.001 | 0.888±0.012 | 0.565±0.002 | 0.610±0.014 | 0.701±0.015 |
| p'=p, n'=0.35n | 0.959±0.008 | 0.922±0.004 | 0.733±0.008 | 0.912±0.010 | 0.619±0.004 | 0.582±0.023 | 0.625±0.028 |
| p'=p, n'=0.17n | **0.971±0.008** | **0.951±0.004** | **0.857±0.013** | **0.923±0.006** | **0.842±0.015** | **0.800±0.013** | **0.875±0.007** |

Table S6. The performance of different data preprocessing method on DAVIS dataset by undersampling

| Samples | AUPRC | AUROC | F1-Score | Sen. | Spec. | Precision | Accuracy |
| --- | --- | --- | --- | --- | --- | --- | --- |
| p=1,808  n=23,964  (original) | 0.900±0.004 | 0.850±0.021 | 0.715±0.009 | 0.810±0.011 | 0.666±0.017 | 0.701±0.015 | 0.596±0.012 |
| p'=p, n'=0.9n | 0.896±0.001 | 0.850±0.002 | 0.712±0.013 | 0.822±0.013 | 0.600±0.021 | 0.715±0.011 | 0.701±0.009 |
| p'=p, n'=0.75n | 0.921±0.007 | 0.866±0.018 | 0.557±0.005 | 0.822±0.003 | 0.781±0.014 | 0.780±0.006 | 0.695±0.019 |
| p'=p, n'=0.60n | 0.990±0.001 | 0.961±0.014 | 0.666±0.015 | 0.833±0.022 | 0.846±0.012 | 0.841±0.012 | 0.751±0.021 |
| p'=p, n'=0.45n | 0.979±0.009 | 0.931±0.005 | 0.727±0.023 | 0.833±0.013 | 0.998±0.008 | 0.979±0.001 | 0.888±0.002 |
| p'=p, n'=0.30n | 0.982±0.012 | 0.935±0.011 | 0.769±0.003 | 0.833±0.017 | 0.923±0.016 | 0.930±0.003 | 0.842±0.009 |
| p'=p, n'=0.15n | 0.996±0.001 | 0.989±0.015 | 0.875±0.003 | **0.875±0.010** | 0.958±0.014 | 0.949±0.001 | 0.851±0.008 |
| p'=p, n'=0.07n | **0.993±0.001** | **0.992±0.001** | **0.896±0.011** | 0.812±0.005 | **0.998±0.001** | **0.998±0.001** | **0.906±0.011** |

## 2.4 High-order graph attention convolutional network interaction prediction module based on structure data

The high-order graph attention network mainly consists of a graph attention network, an encoder and a prediction unit. Each module is described in the supplementary.


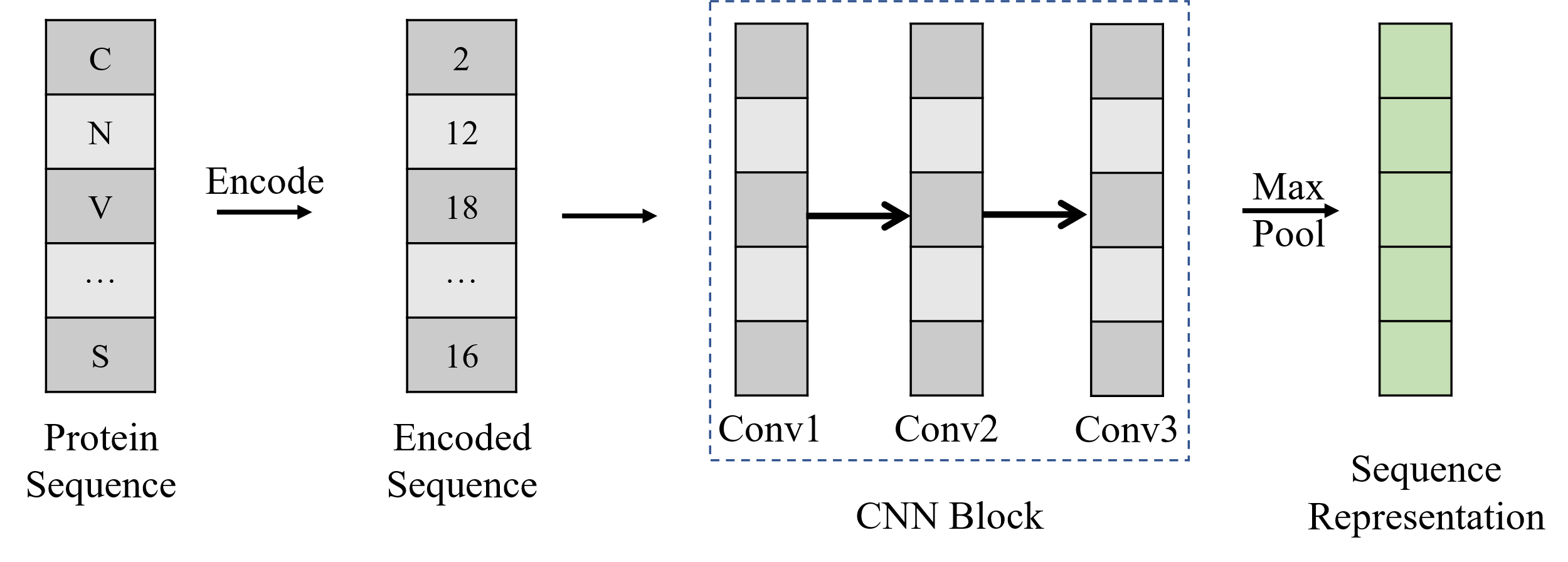


**Fig. S2.** Structure diagram of convolutional neural network.


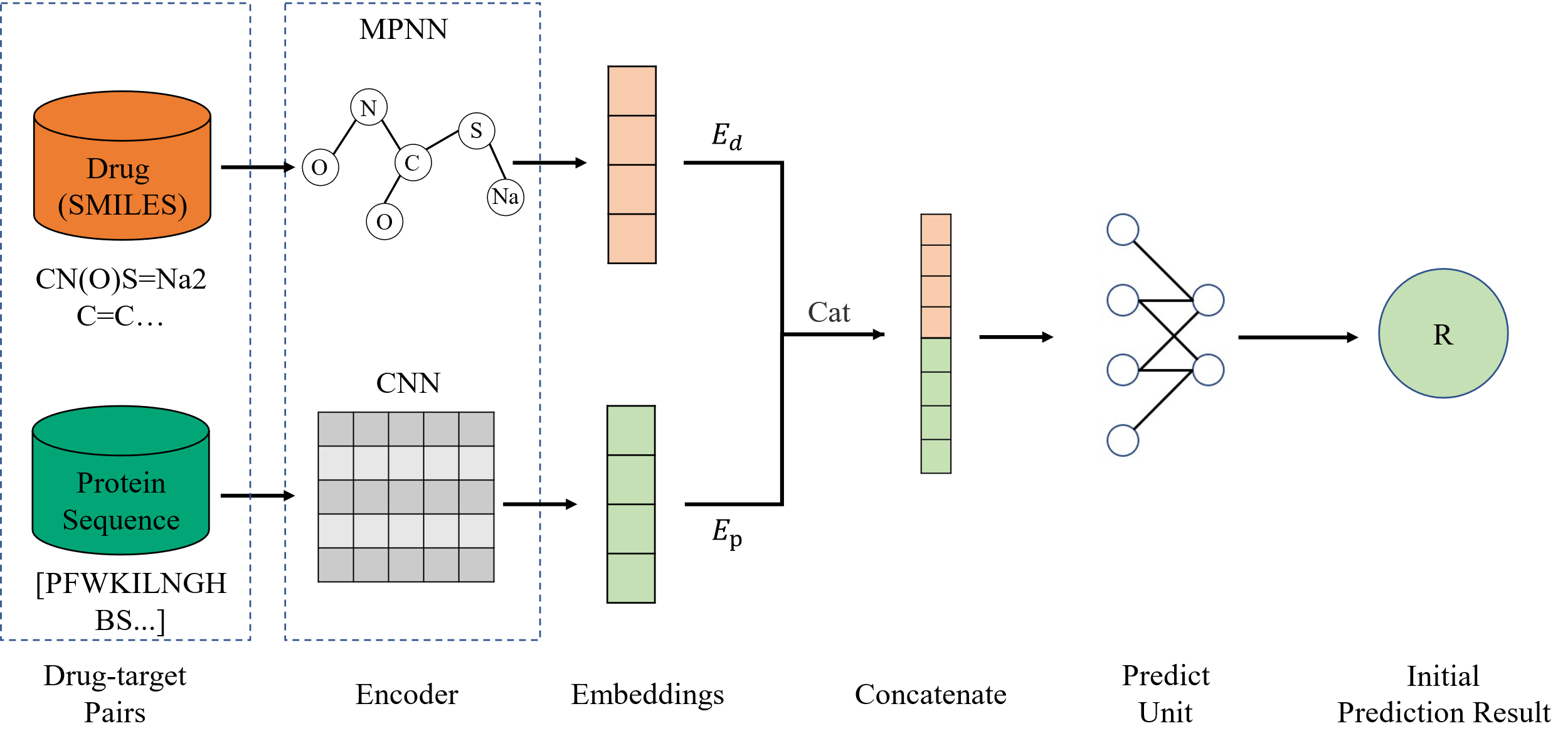


**Fig. S3**. Hybrid deep network for drug-target interaction prediction.

**Graph Attention Network**

The Graph Attention Network (GAT) (Veličković et al. 2018) was used to learn the neighborhood features with attention coefficients in the drug-target relationship graph.

First, the attention coefficient is calculated, given an undirected graph $G$, $i$ is a node in $G$. $H$ denotes the node feature set, $H=\left\{ h_{1},h_{2},\ldots,h_{i} \right\},h_{i}\in R^{F}$. For node $i$, the similarity coefficient between it and its neighbor node $j$ is calculated as follows:

$s_{ij}=f\left(\left[Wh_{i}||Wh_{j}\right]\right)$ (2)

where $W$ is the weight matrix, || denotes the concatenation of the transformed features of nodes $i$, $j$, and $f$ is a single-layer neural network.

After the similarity coefficient $s_{ij}$ is obtained, the attention coefficient $a_{ij}$ is then calculated using equation (2):

 (3)

where $N_{i}$ is the set of nodes $i$ and its neighboring nodes. After obtaining the attention coefficients $\alpha_{ij}$, $k$ attention coefficients are used to perform the calculation ($k=3$ in this study), and finally the new feature $h_{i}^{'}$ of node $i$ containing the neighborhood information is obtained as follows:

 (4)

where || denotes the concatenation, $\alpha_{ij}^{k}$ denotes the kth attention coefficient, $W^{k}$ is the weight matrix, and $\sigma(\cdot)$ is the activation function.

**HOAGCN Encoder**

With the graph attention network in the previous section, features $h$ incorporating neighborhood information are obtained and next the high-order graph attention graph convolution layer (HOAGC) is used as an encoder to process these features and the features $h$ are defined in the form of equation (4):

$H^{(l)}=\mathop{||}\limits_{d\in P} \sigma\left(\hat{A}^{d}H^{(l-1)}W_{d}^{(l)}\right)$ (5)

where $P$ is the set of neighborhood orders, $P=\{1,2,3,\cdot\cdot\cdot,d\}$, which is equivalent to GAT when $d=1$. $\sigma\left( \cdot\right)$ denotes the activation function, $\hat{A}$ is the symmetric normalized adjacency matrix, $\hat{A}=D^{-\frac{1}{2}}\left( A+I_{\left| V \right|} \right)D^{-\frac{1}{2}}$, where $D$ is the diagonal matrix of the graph, $A$ denotes the adjacency matrix, $I$is the unit matrix, $V$ is the number of nodes in the graph, and $\hat{A}^{d}$ denotes the $d$-th power of the adjacency matrix $\hat{A}^{d}$. l is then the number of layers of HOAGC, $W$ is the weight matrix of layer $l$, and ‖ denotes the concatenation.

Fig. S4 shows the specific structure of the high-order attention convolution layers of different orders. The features of different orders in layer $l$ are stitched together to obtain the aggregated features $h^{(l)}$. Blue $h^{(l-1)}$ denotes the features of the previous layer, orange $\hat{A}$ denotes the adjacency matrix, green $W^{(l)}$ denotes the weight matrix, and red $W^{(l)}$ denotes the output features.


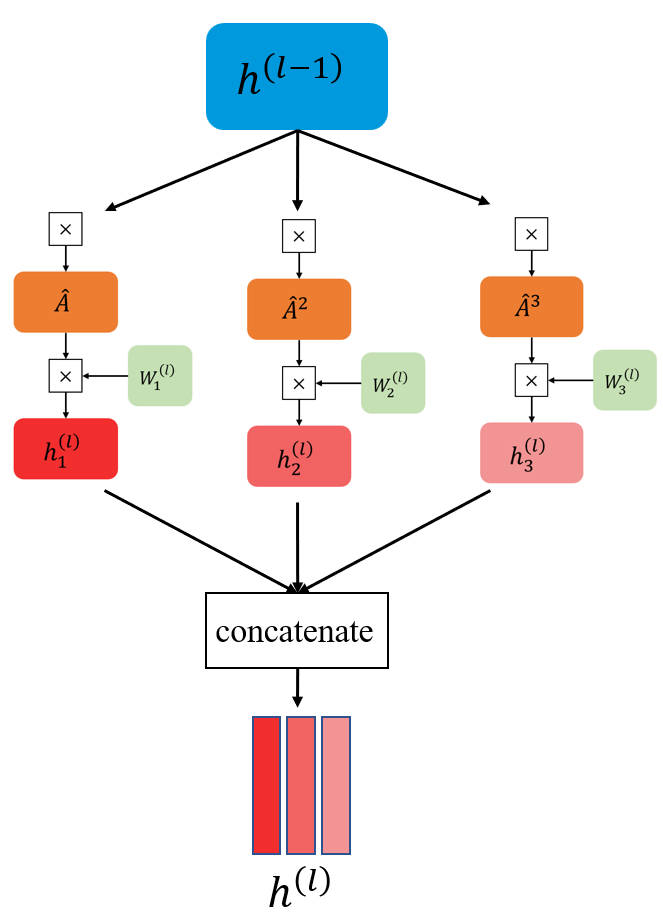


**Fig. S4.** HOAGCN convolution layers.

**HOAGCN Prediction Unit**

With the encoder, the neighborhood aggregation features $h^{\left( l \right)}$ are obtained from different orders. In this part, we use these features to reconstruct the drug-target interactions in the graph. A bilinear layer is used to fuse the aggregated features $h_{i}^{\left( l \right)}$ and $h_{j}^{\left( l \right)}$ of nodes $i$ and $j$, and to learn the features $e_{ij}$ of the edges using equation (5).

$e_{ij}=ELU\left(h_{i}^{\left(l\right)}W_{b}h_{j}^{\left(l\right)}+b\right)$ (6)

where $W$ is the weight matrix, $b$ is the bias, and ELU is the nonlinear activation function. The computed $e_{ij}$ is then fed into a 2-layer fully connected neural network to predict the connection result $P_{ij}$ of nodes $i$ and $j$.

$P_{ij}=sigmoid\left(FC_{2}\left(ELU\left(FC_{1}\left(e_{ij}\right)\right)\right)\right)$ (7)

where $FC_{1}\left( e_{ij} \right)=\boldsymbol{W}_{1}\cdot e_{ij}+\boldsymbol{b}_{1}$ denotes the fully connected layer with weight matrix $\boldsymbol{W}_{1}$ and deviation $\boldsymbol{b}_{1}$, and $FC_{2}$ has the same structure as $FC_{1}$.

**HOAGCN training process**

In the HOAGCN training process, the binary cross entropy loss was used to optimize the model parameters, which is calculated as follows:

$L\left(v_{i},v_{j}\right)=-A_{ij}log\left(P_{ij}\right)-\left(1-A_{ij}\right)log\left(1-P_{ij}\right)$ (8)

where $P_{ij}$ is the predicted interaction probability between $v_{i}$ and $v_{j}$, and $A_{ij}$ denotes the true interaction labels between nodes $i$ and $j$. Considering all interactions, the final loss function obtained by summing Equation 8:

$L=\sum_{\left(i,j\right)\in E} L\left(v_{i},v_{j}\right)$ (9)

An end-to-end approach was used to jointly optimize all trainable parameters and back-propagate the gradients through the encoder and prediction unit of HOAGCN. The whole training process is shown in Algorithm 1.


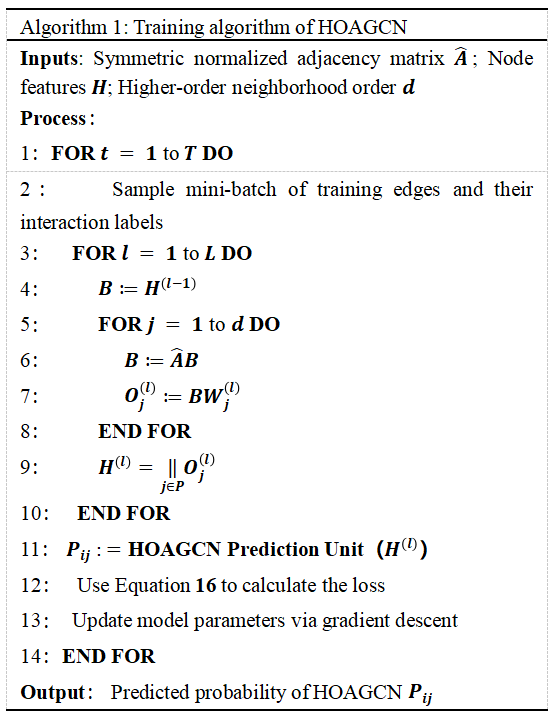


## 2.5 Evaluation metrics

To evaluate the performance of the proposed method, five evaluation metrics: sensitivity (Sen.), specificity (Spec.), F1-Score (F1), area under the ROC curve (AUROC), and area under the PRC curve (AUPRC) were used in this study. The range of all above metrics is [0, 1], and higher values of all metrics better performance. The specific definitions of the above metrics are as follows:

$Sen.=\frac{TP}{TP+FN}$ （10）

$Spec.=\frac{TN}{TP+FN}$ （11）

$F1=\frac{2TP}{2TP+FP+FN}$ （12）

$$\begin{aligned} Precision=\frac{TP}{TP + FP}\#\left( 13 \right) \end{aligned}$$

$$\begin{aligned} Accuracy=\frac{TP+TN}{TP + FP+TN+FN}\#\left( 14 \right) \end{aligned}$$

where $TP$ is the number of true positive samples, $TN$ is the number of true negative samples, $FP$ is the number of negative samples misidentified as positive samples, and $FN$ means the number of positive samples misidentified as negative samples. The F1-score is used to combine precision and recall metrics, which better assesses the predictive performance of unbalanced data.

By calculating $\mathrm{TPR}={TP}/{(TP+FN)}$ and $\mathrm{FPR}={FP}/{(FP+TN)}$, the receiver operating characteristic (ROC) curve can be determined, and it can determine the trade-off between TPR and FPR, and then the AUROC value can be calculated. In addition, the AUPRC value can be obtained by using the precision and recall rates. In the case of data imbalance, AUPRC can be obtained to evaluate the effect more accurately than AUROC.

## 2.6 Case analysis

To further evaluate the performance of the proposed method in this study, we performed the following analysis of the prediction results: the existing drug-target interactions in the BindingDB dataset were predicted using MINDG and given binding affinity values. Based on the prediction results, we extracted five new connections with high binding affinity values that were not previously recorded in the database, as shown in Table S7. Meanwhile, we verified the prediction results on DrugBank based on the searched drug-target interactions. The DrugBank database contains 9591 drug entries and includes 2037 FDA-approved small molecule drugs, 241 FDA-approved biotechnology (protein/peptide) drugs, 96 nutraceuticals, and more than 6000 experimental drugs, in addition to 4270 sequence associations of non-redundant proteins (i.e., targets, enzymes, transport proteins, and carriers). These data can provide the necessary proof to support our predicted results.

As shown in Fig. S5, Clonazepam, for example, is a long-acting benzodiazepine with a moderate onset of action and is commonly used to treat panic disorder, severe anxiety disorder, and seizures. MINDG captures the interaction between clonidine and the target Prothrombin. The database shows that Prothrombin also interacts with other drugs used to treat psychiatric problems, such as ioperidone, trimipramine, and ergoloid. Since all of the above drugs are relevant to the genesis and treatment of psychiatric disorders, this could support the demonstration that the target Prothrombin has the potential to interact with clonidine in a real way. The above analysis shows that our predicted results are favorably supported by the findings of the relevant literature. Although there are still some predicted drug-target pairs that are not supported by the DrugBank database, the possibility of an association between them cannot be denied, which needs to be verified by further biological experiments.

Table S7 New connections predicted by MINDG

| Drug | Target | Binding Affinity |
| --- | --- | --- |
| Moclobemide | Cytochrome P450 3A5 | 7.2169 |
| Clonazepam | Prothrombin | 6.4813 |
| loperidone | Myeloperoxidase | 5.9465 |
| Palonosetron | Glycine | 4.8983 |
| Netupitant | ATP | 4.6214 |


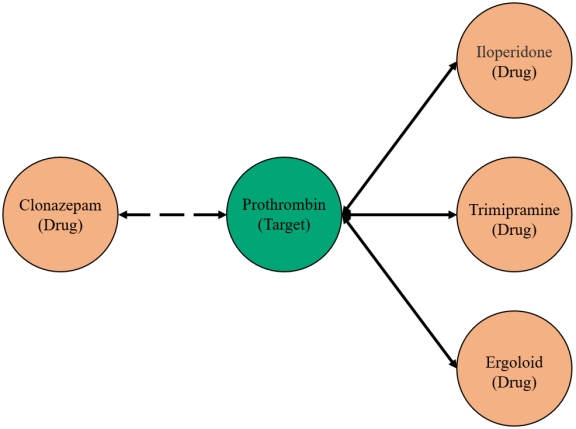


**Fig. S5.** Predicted interaction between the drug Clonazepam and the target. The solid black line is known interactions and the dashed line indicates new interactions discovered by MINDG.

## 2.7 The impact of multi-view adaptive integrated decision module on prediction performance

In this section, the effectiveness of the multi-view adaptive integrated decision module used by MINDG is verified. To evaluate the performance of the adaptive integrated decision module, the corresponding version that directly uses simple arithmetic average of multi-view results is defined as MINDG_avg in the equation (15) and then compared with MINDG. The experimental results are shown in the supplementary (Fig. S6), and it can be seen that MINDG outperforms MINDG_avg in every metric on both the BindingDB and DAVIS datasets. This also indicates that the adaptive multi-view integrated decision making using multi-view adaptive weighting mechanism has some advantages over the traditional simple averaging strategy.

$$\begin{aligned} MINDG_{avg}=\frac{1}{V}\sum_{v=1}^{V} R_{v}\#\left( 15 \right) \end{aligned}$$

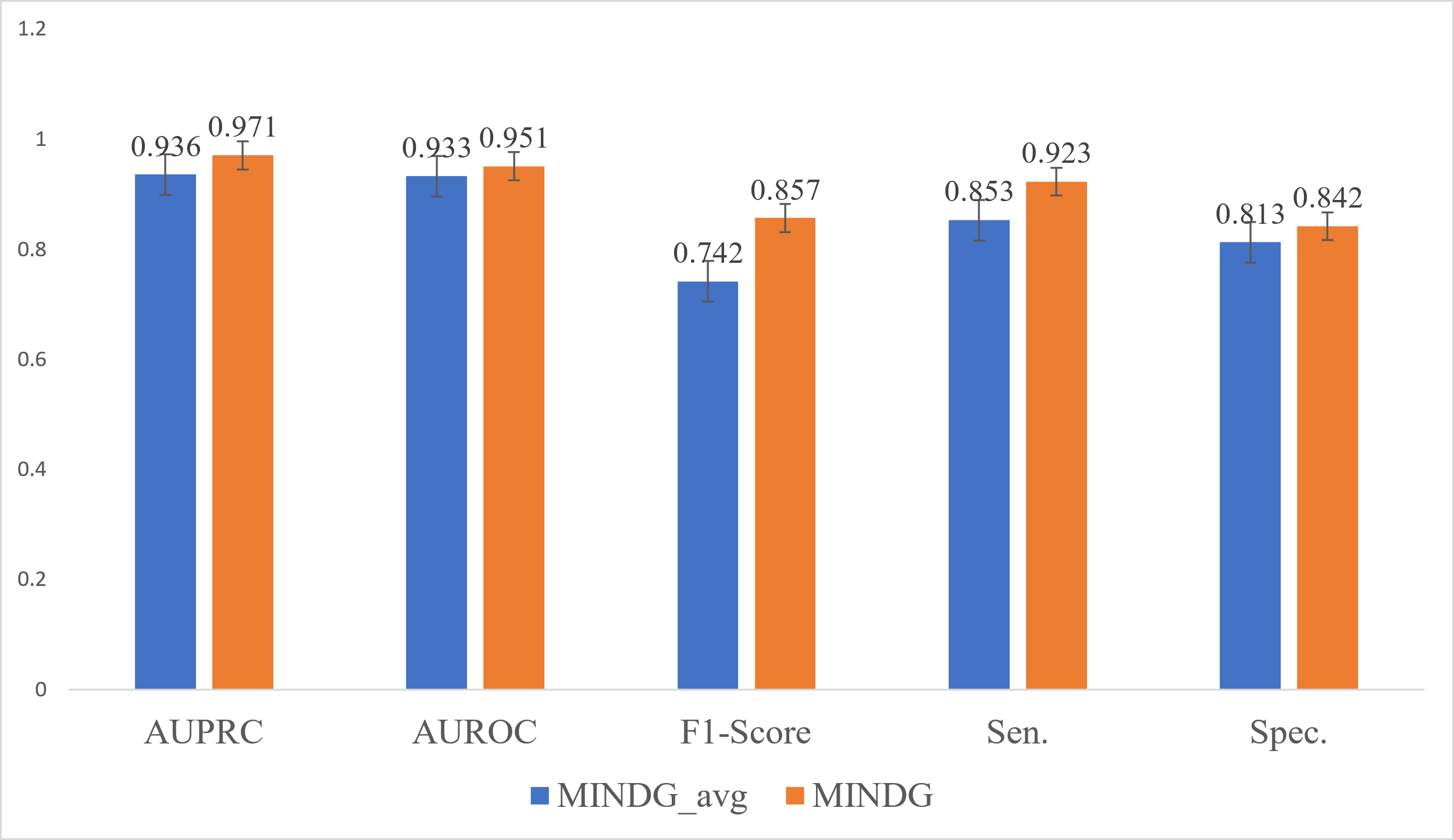


（A）


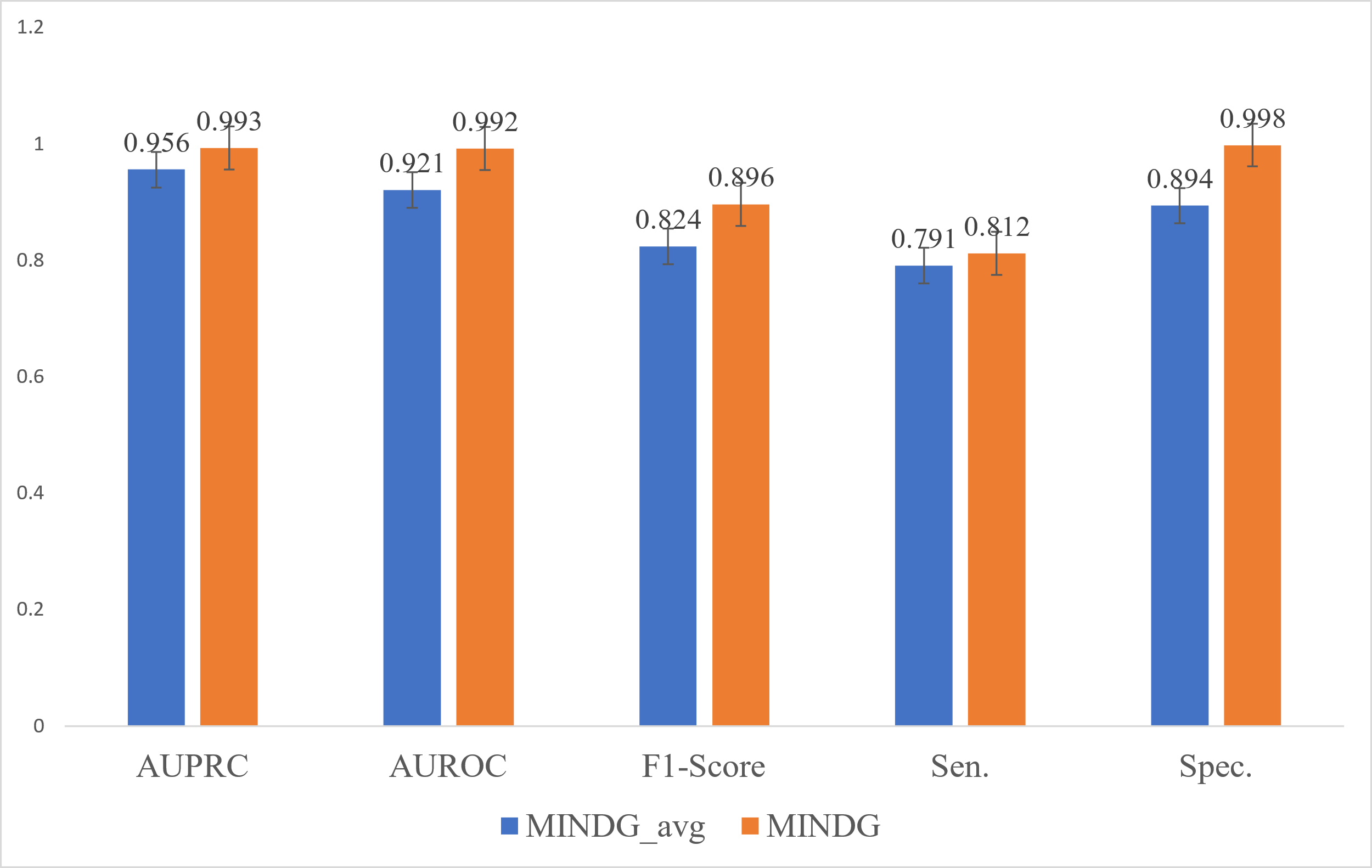


(B)

**Fig. S6.** (A)Validity assessment of multi-view adaptive integrated decision module.on BindingDB (B) Validity assessment of multi-view adaptive integrated decision module.on DAVIS

## 2.8 Impact of high-order neighborhoods on prediction performance

In this section, the effect of high-order neighborhood order on the performance of the model is verified with different neighborhood orders $d$ to observe the performance variation of the proposed method MINDG, and the experimental results are shown in Fig. S7 shows the AUROC metrics obtained by MINDG on the BindingDB and DAVIS datasets with different neighborhood orders $d=\{1,2,3,4,5\}$. When $d=1$, the network is comparable to GAT at this point, and as $d$ increases, the performance of MINDG becomes better, further demonstrating the effectiveness of MINDG in aggregating high-order neighborhood features. The results show that for all data sets, the performance of MINDG works best at order 3. Starting from order 4, the model starts to be insensitive to $d$, and MINDG achieves similar performance when $d=\{4,5\}$. The above analysis shows that the 3rd order neighborhood already provides enough information for interaction prediction, at which point the performance remains relatively stable and difficult to improve further even if the value of d increases further.


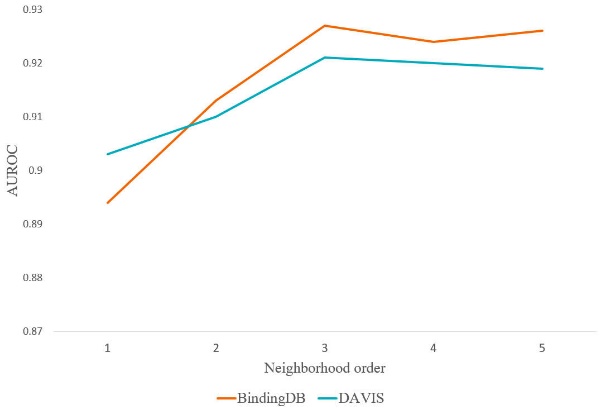


**Fig. S7** Effect of different order neighborhoods on the prediction performance of MINDG.

## 2.9 Significance and Sensitivity Test

Statistical significance was tested for the results of all methods (DeepCDA, TripletMultiDTI, GAT, MINDG) using the Friedman Method (Pereira et al., 2015). Table 8 shows that all p-values are less than the significance level (α = 0.05), indicating statistically significant differences between the results of all methods.

Table S8 Friedman Test of different methods on BindingDB and DAVIS datasets

| Dataset | statistic | p-value |
| --- | --- | --- |
| BindingDB | 10.920±0.001 | 0.012±0.001 |
| DAVIS | 14.039±0.005 | 0.003±0.001 |

The binding affinity value indicates the strength of interaction between drugs and protein targets. The predicted labels for all methods are derived from the binarization of binding affinity values. To verify the sensitivity of the DTI results, we used MINDG as an example and obtained the performance curves shown in Fig. S8 by adjusting the binding affinity threshold to {5, 6, 7, 8, 9, 10}. The curves show that the primary outcomes of MINDG are influenced by the drug-target interaction used for performance comparison.


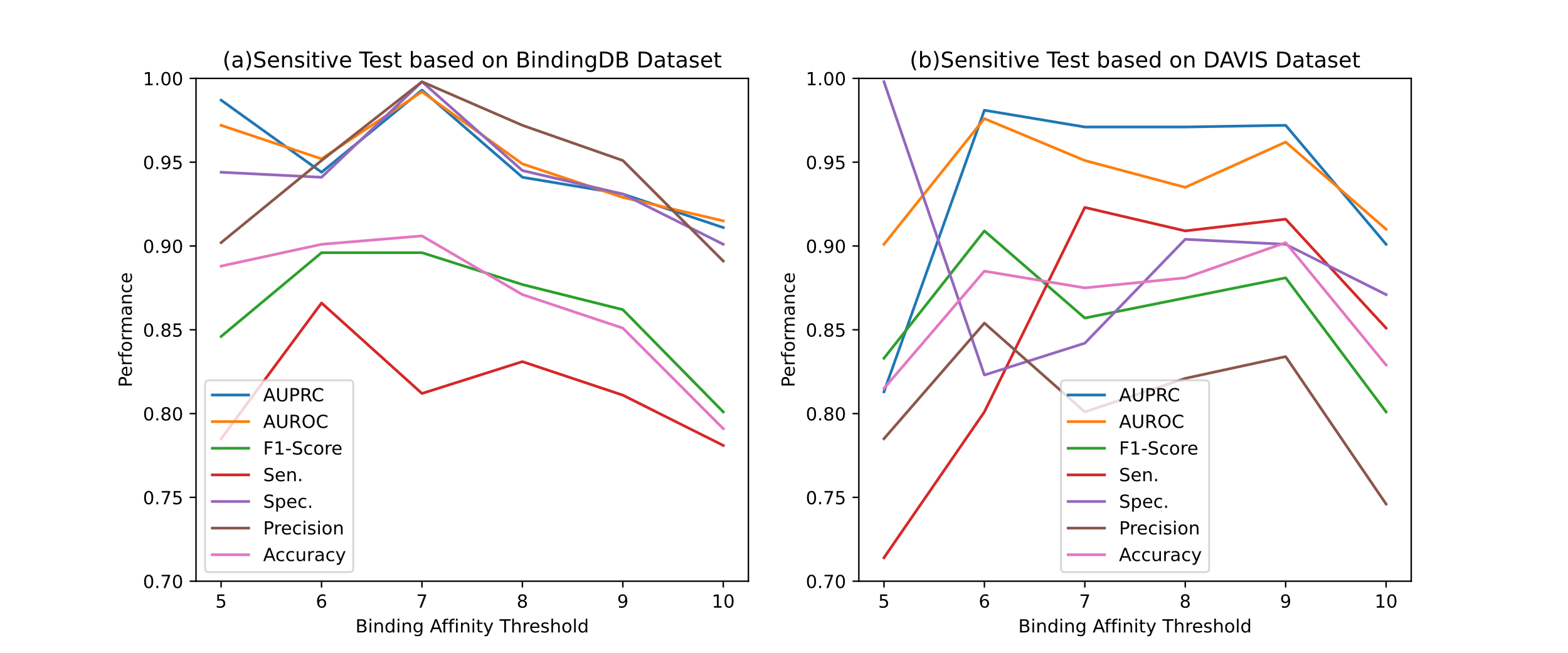


**Fig. S8**. Sensitive Test based on BindingDB and DAVIS dataset

## 2.10 Analysis of model calibration performance

This section validates the calibration performance of the model, specifically the confidence estimates of the drug-target interactions. The aim is to test whether the predicted confidence level represents the probability of a true interaction. In other words, the calibrated confidence estimates should be representative of the true interaction probability (Niculescu-Mizil et al. 2005). For instance, if we have 100 predictions, each with a confidence of 0.9, we can expect 90 interactions to be correctly classified as true interactions.

To assess the calibration performance, we utilized reliability plots and Brier scores (Brier 1950). The reliability diagrams offer a clear visual representation of the model calibration by dividing the samples into 10 buckets based on the predicted probability of the model output. This means that samples with predictions ranging from 0 to 0.1 are grouped into one bucket, and so on. To ensure clear representation, we use the average predictive value as horizontal coordinates for these 10 buckets. The vertical coordinate represents the percentage of positive samples within each bucket, and a diagonal line represents the model with perfectly calibrated predictions. The Brier score is a suitable scoring rule for measuring the accuracy of prediction probabilities. A lower Brier score indicates better calibration of predictions. It is calculated as the mean squared error of the predicted probability $P_{ij}$ and the ground-truth interaction label $A_{ij}$. Mathematically, the Brier score can be expressed using equation (14):

$$\begin{aligned} \text{Brier} \text{score} =\frac{1}{N}\sum_{\left( i,j \right)=1}^{N} \left( P_{ij}-A_{ij} \right)^{2}\#\left( 16 \right) \end{aligned}$$

where $N$ denotes the number of edges (interactions).

Fig. S9 and Fig. S10 show the calibration diagrams of MINDG on the BindingDB and DAVIS datasets, respectively. As can be seen in Fig. S9, MINDG achieves better calibration performance on the BindingDB dataset, while DeepDTA is relatively less confident. For example, in the interaction with a prediction confidence of 0.6, DeepDTA has a positive sample share of 75%, which is 15 % higher. At this point, DeepDTA's classification accuracy is higher than its confidence level, indicating that the model is not confident in its prediction. It can be seen from Fig. S10 that GAT and GCN achieve better calibration performance compared to MINDG, indicating that learning of first-order neighborhood information is more appropriate in the DAVIS dataset, and aggregating features from high-order neighborhoods would make the model too confident. And DeepDTA remains relatively less confident in the DAVIS dataset.


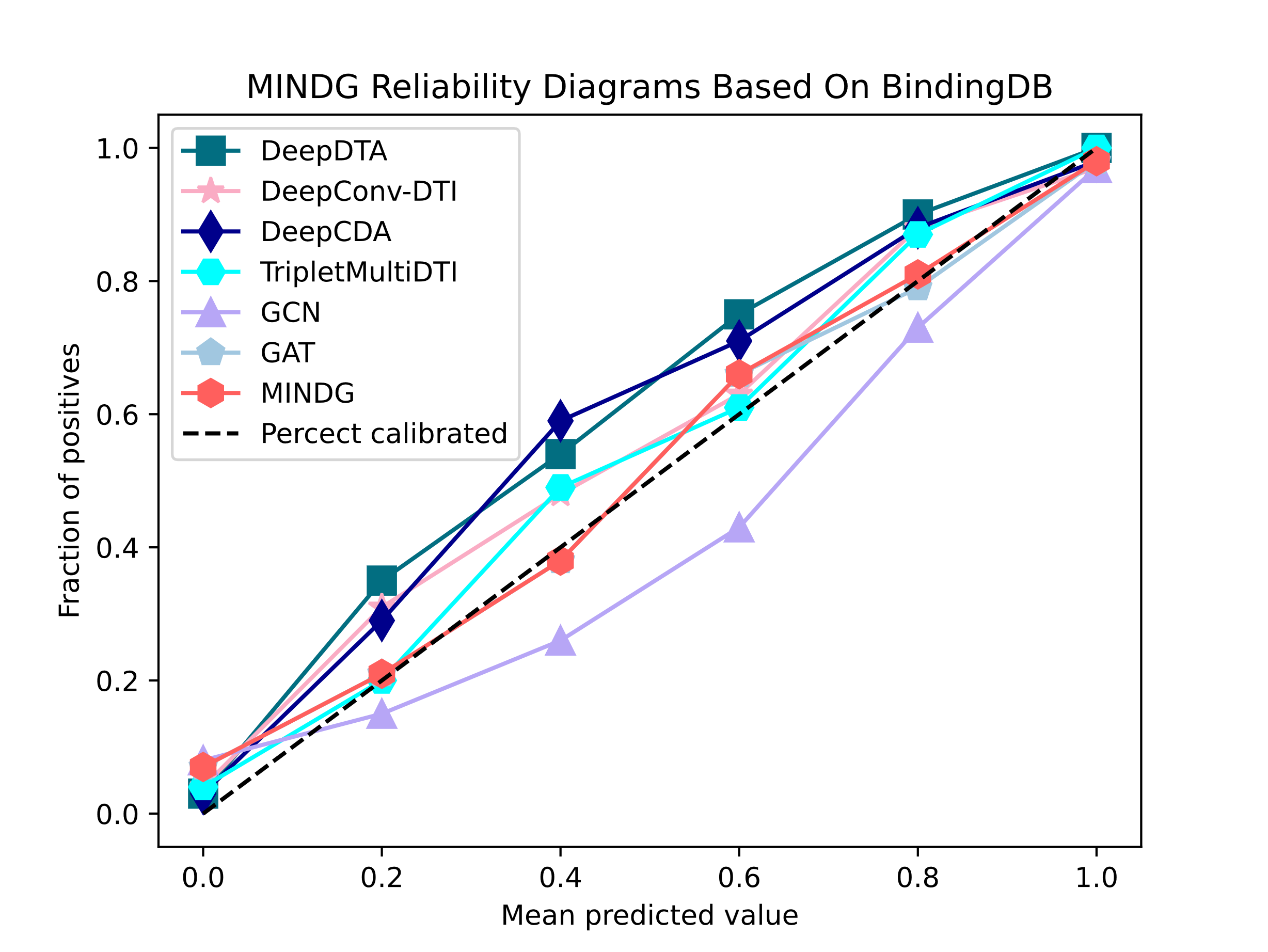


**Fig. S9**. Performance evaluation of model calibration based on BindingDB dataset.


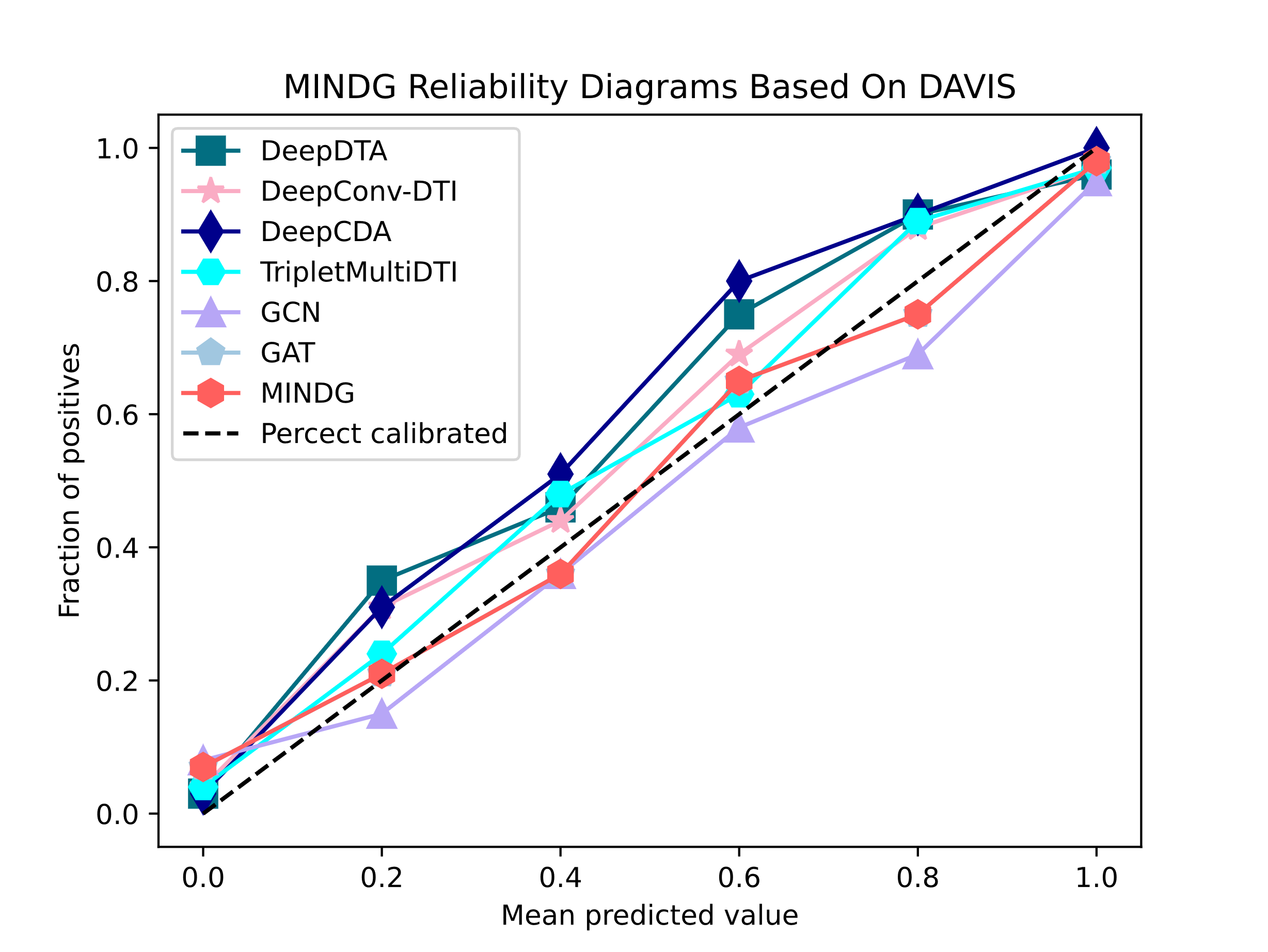


**Fig. S10**. Performance evaluation of model calibration based on DAVIS dataset.

## 2.11 Repurposing of antiviral drugs for COVID-19 targets

In addition, based on the target SARS-CoV-2 3CL protease of the COVID-19, we used MINDG for the exploration of antiviral drug repurposing. Using the SARS-CoV-2 3CL protease sequence resolved by Gao et al.( Gao et al. 2020) input into the model, the top 10 drugs with binding affinity values were predicted as shown in Table S9:

Table S9 Results of drug repurposing of SARS-CoV2 3CL protease

| Rank | Drug | Binding Affinity | Rank | Drug | Binding Affinity |
| --- | --- | --- | --- | --- | --- |
| 1 | Foscarnet | 16.365 | 6 | Rilpivirine | 8.432 |
| 2 | Favipiravir | 14.489 | 7 | Sofosbuvir | 6.891 |
| 3 | Arbidol | 13.241 | 8 | Glecaprevir | 6.230 |
| 4 | Remdesivir | 13.102 | 9 | Rimantadine | 4.459 |
| 5 | Rimantadine | 10.039 | 10 | Amantadine | 3.187 |

Coronaviruses begin to replicate and clone in large numbers once they have invaded cells. In turn, the viral RNA-dependent RNA polymerase (RdRp, also known as nsp12) plays a crucial role in the synthesis of the genetic material of the virus, RNA. As the core component of this replication machine, RNA polymerase is one of the most important antiviral targets, as it is the key to viral reproduction in large numbers. By disrupting RNA polymerase, the self-replication and number expansion of the virus can be stopped, achieving the ultimate therapeutic goal.

Among the predicted results in Table 2, fapiravir (Seneviratne et al. 2020) is currently undergoing a global multicenter clinical trial for the treatment of coronavirus. Published clinical data suggest that the drug can rapidly clear the virus and achieve relief of COVID-19 symptoms, accompanied by fewer adverse effects and higher tolerability. In February 2020, favipiravir was used in China for the experimental treatment of COVID-19 (Li et al. 2020). On March 17, the drug was found to be effective in the treatment of infected patients in trials conducted in Wuhan and Shenzhen (Xinhua 2022). The drug ranked third in terms of binding affinity, Arbidol (Boriskin et al. 2008), is an antiviral drug, developed by the Pharmaceutical Chemistry Research Center in the Soviet Union, with the main indication of influenza caused by influenza A and B viruses, and may also have antiviral activity against some other respiratory viral infections. The fourth ranked drug, remdesivir (Nhean et al. 2021), is a prodrug (de Albuquerque Silva et al. 2005), (Han et al. 2000) biotransformed into a ribonucleotide analogue inhibitor capable of inhibiting the viral RNA polymerase. Therefore, remdesivir is considered a highly promising clinical agent for the treatment of COVID-19. On October 22, 2020, the US Food and Drug Administration approved raltegravir as the first drug for the treatment of COVID-19. In combination with Foscarnet, the drug with the top affinity value, and Efavirenz itself, both drugs for the treatment of AIDS (HIV) ( Canestri et al. 2006; Meyer et al. 2007; Stellbrink 2007), are non-nucleoside reverse transcriptase inhibitors that also have an inhibitory function on RNA polymerase, a property captured by MINDG. Rimantadine (Wintermeyer et al. 1995) is an antiviral agent belonging to the tricyclic amine class with prophylactic and therapeutic effects against influenza A virus, exerting antiviral activity or inhibiting virus-specific RNA synthesis by inhibiting reverse transcriptase. Glecaprevir (Lamb 2017) is a direct-acting antiviral agent and NS3/4A of hepatitis C virus (HCV) protease inhibitor, which also targets the inhibition of viral RNA replication.

The above case studies and practical applications of drug efficacy in MINDG-generated candidate drug lists help demonstrate the informative value of MINDG prediction results.

# Reference

Albuquerque Silva AT, Chung MC, Castro LF, Carvalho Guido RV, Ferreira EI. Advances in prodrug design. *Mini reviews in medicinal chemistry*, 2005; **5**:893-914.

Brier GW. Verification of forecasts expressed in terms of probability. *Monthly weather review* 1950; **78**:1-3.

Biau G, Scornet E. A random forest guided tour. *Test*, 2016; **25**:197-227.

Boriskin YS, Leneva IA, Pecheur EI, Polyak SJ. Arbidol: a broad-spectrum antiviral compound that blocks viral fusion. *Current medicinal chemistry*, 2008; **15**:997-1005.

Canestri A, *et al.* Foscarnet Salvage Therapy for Patients with Late-Stage HIV Disease and Multiple Drug Resistance. *Antiviral Therapy*, 2006; **11**:561-566.

Han HK, Amidon GL. Targeted prodrug design to optimize drug delivery. *Aaps Pharmsci*, 2000; **2**: 48-58.

He T, Heidemeyer M, Ban F, et al. Simboost: a read-across approach for predicting drug–target binding affinities using gradient boosting machines. *Journal of Cheminformatics* 2017; **9**:24.

Hinton GE, Osindero S, Teh YW. A fast learning algorithm for deep belief nets. *Neural computation*. 2006; **18**:1527-1554.

Gao Y, *et al.* Structure of RNA-dependent RNA polymerase from 2019-nCoV, a major antiviral drug target. *BioRxiv*, 2020; 2020-2030.

Lamb YN. Glecaprevir/pibrentasvir: first global approval. *Drugs*, 2017; **77**:1797-1804.

Lee I, Keum J, Nam H. DeepConv-DTI: Prediction of drug-target interactions via deep learning with convolution on protein sequences. *PLoS computational biology*. 2019; **15**:e1007129.

Li G, De Clercq E. Therapeutic options for the 2019 novel coronavirus (2019-nCoV). *Nature reviews Drug discovery*, 2020; **19**:149-150.

Meyer PR, Rutvisuttinunt W, Matsuura SE, So AG, Scott WA. Stable Complexes Formed by HIV-1 Reverse Transcriptase at Distinct Positions on the Primer-Template Controlled by Binding Deoxynucleoside Triphosphates or Foscarnet. *J Mol Biol*, 2007; **369**:41-54.

Nagamine N, Sakakibara Y. Statistical prediction of protein–chemical interactions based on chemical structure and mass spectrometry data. *Bioinformatics*, 2007; **23**:2004-2012.

Nhean S *et al.* COVID-19: a review of potential treatments (corticosteroids, Remdesivir, tocilizumab, bamlanivimab/etesevimab, and casirivimab/imdevimab) and pharmacological considerations. *Journal of pharmacy practice*, 2021; 08971900211048139.

Pereira DG, Afonso A, Medeiros FM. Overview of Friedman’s Test and Post-hoc Analysis. *Communications in Statistics - Simulation and Computation* 2015; **44**: 2636–2653.

Öztürk H, Ozkirimli E, Özgür A. DeepDTA: Deep Drug-Target Binding Affinity Prediction. Bioinformatics, 2018; 34: i821–i829.

Rodriguez JD, Perez A, Lozano JA. Sensitivity Analysis of k-Fold Cross Validation in Prediction Error Estimation. *IEEE Trans. Pattern Anal. Mach. Intell.* 2009; **32**: 569–575.

Seneviratne SL, Abeysuriya V, De Mel S, De Zoysa I, Niloofa R. Favipiravir in COVID-19. *International Journal of Progressive Sciences and Technologies*, 2020; **19**:143-145.

Shao K, Zhang Z, He S, Bo X. DTIGCCN: Prediction of drug-target interactions based on GCN and CNN. *2020 IEEE 32nd International Conference on Tools with Artificial Intelligence (ICTAI)*, 2020:337-342.

Shi H, Liu S, Chen J, Li X, Ma Q, Yu B. Predicting drug-target interactions using Lasso with random forest based on evolutionary information and chemical structure. *Genomics*, 2019; **111**:1839-1852.

Stellbrink HJ. Antiviral drugs in the treatment of AIDS: what is in the pipeline. *Eur J Med Res*, 2007; **12**:483-495.

Öztürk H, Ozkirimli E, Özgür A. DeepDTA: Deep Drug-Target Binding Affinity Prediction. *Bioinformatics*, 2018; **34**:i821–i829.

Thafar MA, *et al.* DTi2Vec: Drug–target interaction prediction using network embedding and ensemble learning. *J Cheminform*, 2021; **13**:1.

Veličković P, Cucurull G, Casanova A, Romero A, LiòP, Bengio Y. Graph Attention Networks. arXiv, 2018.

Wang H, Zhou G, Liu S, Jiang JY, Wang W. Drug-Target Interaction Prediction with Graph Attention networks. arXiv, 2021.

Wang L, *et al.* A computational-based method for predicting drug–target interactions by using stacked autoencoder deep neural network. *Journal of Computational Biology*, 2018; **25**:361-373.

Wintermeyer SM, Nahata MC. Rimantadine: a clinical perspective. *Annals of Pharmacotherapy*, 1995; **29**:299-310.

Wishart DS, *et al.* DrugBank 5.0: a major update to the DrugBank database for 2018. *Nucleic acids research*, 2018; **46**:D1074–D1082.

Wu F, Souza A, Zhang T, Fifty C, Yu T, Weinberger K. Simplifying graph convolutional networks. *International conference on machine learning*, PMLR, 2019; 6861-6871.

Xinhua P. Favipiravir shows good clinical efficacy in treating COVID-19: official-Xinhua | English.news.cn. <http://www.xinhuanet.com/english/2020-03/17/c_138887971.htm>, 2022.

Yu H, *et al.* A systematic prediction of multiple drug-target interactions from chemical, genomic, and pharmacological data. *PloS one*, 2012; **7**:e37608.
